# Examination of the interaction between age‐specific predation and chronic disease in the Greater Yellowstone Ecosystem

**DOI:** 10.1111/1365-2656.13661

**Published:** 2022-01-27

**Authors:** Ellen E. Brandell, Paul C. Cross, Douglas W. Smith, Will Rogers, Nathan L. Galloway, Daniel R. MacNulty, Daniel R. Stahler, John Treanor, Peter J. Hudson

**Affiliations:** ^1^ Center for Infectious Disease Dynamics and Department of Biology, Huck Institutes of the Life Sciences Pennsylvania State University University Park PA USA; ^2^ Wisconsin Cooperative Wildlife Research Unit, Department of Forest and Wildlife Ecology University of Wisconsin‐Madison Madison WI USA; ^3^ U.S. Geological Survey Northern Rocky Mountain Science Center Bozeman MT USA; ^4^ Yellowstone Center for Resources Yellowstone National Park Wyoming WY USA; ^5^ Department of Ecology Montana State University Bozeman MT USA; ^6^ Biological Resources Division National Park Service Fort Collins CO USA; ^7^ Department of Wildland Resources Utah State University Logan UT USA

**Keywords:** age structure, demography, healthy herds, infectious disease, matrix model, predator–prey, prion, simulation

## Abstract

1. Predators may create healthier prey populations by selectively removing diseased individuals. Predators typically prefer some ages of prey over others, which may, or may not, align with those prey ages that are most likely to be diseased.
2. The interaction of age‐specific infection and predation has not been previously explored and likely has sizable effects on disease dynamics. We hypothesize that predator cleansing effects will be greater when the disease and predation occur in the same prey age groups.
3. We examine the predator cleansing effect using a model where both vulnerability to predators and pathogen prevalence vary with age. We tailor this model to chronic wasting disease (CWD) in mule deer and elk populations in the Greater Yellowstone Ecosystem, with empirical data from Yellowstone grey wolves and cougars.
4. Model results suggest that under moderate, yet realistic, predation pressure from cougars and wolves independently, predators may decrease CWD outbreak size substantially and delay the accumulation of symptomatic deer and elk. The magnitude of this effect is driven by the ability of predators to selectively remove late‐stage CWD infections that are likely the most responsible for transmission, but this may not be the age class they typically select. Thus, predators that select for infected young adults over uninfected juveniles have a stronger cleansing effect, and these effects are strengthened when transmission rates increase with increasing prey morbidity. There are also trade‐offs from a management perspective—that is, increasing predator kill rates can result in opposing forces on prey abundance and CWD prevalence.
5. Our modelling exploration shows that predators have the potential to reduce prevalence in prey populations when prey age and disease severity are considered, yet the strength of this effect is influenced by predators' selection for demography or body condition. Current CWD management focuses on increasing cervid hunting as the primary management tool, and our results suggest predators may also be a useful tool under certain conditions, but not necessarily without additional impacts on host abundance and demography. Protected areas with predator populations will play a large role in informing the debate over predator impacts on disease.

## Graphical abstract

Predation and disease prevalence are often concentrated in certain prey age classes. The interaction between age‐specific infection and predation has yet to be explored and may have substantial effects on disease dynamics. The authors find that predator cleansing effects are amplified when, within the same age class, there is simultaneous selection by predators and aggregation of infection.

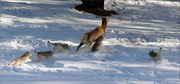

## 1 INTRODUCTION

The patterns of parasite infections in wildlife hosts often have an age component. For example, the prevalence of chronic infections tends to skew towards older individuals that have had a longer amount of time to be exposed (e.g. Heisey et al., 2006). Conversely, intestinal parasite infection prevalence is skewed towards younger individuals (e.g. Cattadori et al., 2005). Predators can improve the health of prey populations by selectively removing infectious individuals (Packer et al., 2003), and their foraging behaviour and impact on prey abundance often vary by prey species, age, sex and condition (e.g. Gervasi et al., 2012; Hoy et al., 2021). Little is known, however, about how age‐specific variation in parasite prevalence within prey populations alters the cleansing effect of predators that prefer certain age groups. While both age‐specific infection and predation are well‐studied, their intersection has not been previously explored and is likely very important for disease dynamics. We hypothesize that the predator cleansing effect will be more efficient in systems where the disease and predation are occurring in the same prey age groups. We test this hypothesis using a mathematical model based on predation and age structure data from cougars *Puma concolor*, wolves *Canis lupus*, elk *Cervus canadensis* and mule deer *Odocoileus hemionus* in Yellowstone National Park.

Chronic wasting disease (CWD), a fatal prion disease, has been associated with population declines in mule deer and elk (DeVivo et al., 2017; Edmunds et al., 2016; Monello et al., 2014), threatening cervid populations in western North America (Mysterud & Edmunds, 2019). As a chronic infection, young to prime‐aged adults are most likely to test positive (Miller & Conner, 2005; Monello et al., 2014). Mule deer tend to have higher prevalence and greater population‐level sensitivity to CWD infection, but CWD can also limit elk population growth (Monello et al., 2014; Sargeant et al., 2011; Williams et al., 2014). Predators have been proposed as a potential biological control mechanism for CWD in cervid populations, and there is some evidence that cougars select for CWD‐infected mule deer (Krumm et al., 2010; Miller et al., 2008). Nevertheless, Miller et al. (2008) found that CWD prevalence in mule deer remained high even under high rates of selective cougar predation.

Here we explore the conditions under which selective predation by cougars and wolves could reduce CWD prevalence in mule deer and elk populations while accounting for host age, selective predation and disease progression. The Greater Yellowstone Ecosystem (GYE) provides an excellent opportunity to study these processes because cervid prey, including elk and mule deer, and predators such as wolves and cougars are intensively monitored year‐round. CWD has been spreading in elk and deer in the GYE since 2018 (Montana Fish, Wildlife, & Parks, 2020; National Park Service, 2018, 2020). In Yellowstone, wolves primarily kill elk, while cougars kill elk and mule deer at approximately equal rates, thus we suspect that cougars will best limit CWD invasion in deer, and wolves in elk (Metz, Hebblewhite, et al., 2020; Stahler et al., 2020).

Predators will reduce CWD when they reduce the growth rate of the infection to a point where the prey population escapes disease limitation (i.e. reducing *R*_0_). This occurs through at least three interacting mechanisms: (a) shortening the life span of infected individuals which reduces the duration of infectiousness, (b) selectively removing the most infectious prey, which reduces transmission rate and (c) decreasing the size of the susceptible prey population, which may reduce the number of potential infectious contacts. More than 25 years of field evidence from the northern GYE shows wolves selectively kill prey in poor body condition, especially senescent adults, neonates and adults with depleted fat reserves (MacNulty et al., 2020; Metz et al., 2012; Metz, Hebblewhite, et al., 2020), whereas cougars select less on body condition and more on body size (Husseman et al., 2003; Ruth et al., 2019). In Yellowstone, cougars primarily prey on juvenile elk and juvenile and adult mule deer (Ruth et al., 2019).

CWD spreads slowly and has only recently invaded areas inhabited by abundant large predators, such as the GYE, so the empirical effects of selective predators on CWD invasion and spread may not be evident for years to decades. In the meantime, models can provide a mechanistic expectation for the GYE and similar ecosystems that can be compared with empirical data in the future. We developed a mechanistic, sex‐ and age‐structured host population model for two types of predator–prey systems: cougar–deer (i.e. mule deer) and wolf–elk, with empirically validated parameters (Appendices S1 and S2). Our model advances previous CWD‐predator models by considering that prey selection is a function of prey age and infection severity. Additionally, our model accounts for the dynamic relationship between the predator and prey populations and the influence of CWD progression on host transmission rates. We present deterministic results of potential scenarios, while acknowledging that there is a high level of uncertainty in many model parameters. Our intent is to use the model to understand parameter interactions rather than create predictions specific to CWD in the GYE. Hence, our research adds to the broader understanding of disease dynamics across multiple host age classes and trophic levels, highlights important ecological considerations when constructing disease models and management protocols and provides guidance for future data collection.

## 2 MATERIALS AND METHODS

We used a deterministic model of cougar–deer and wolf–elk systems that can be parameterized for other systems (Equation 1). Below we summarize our model's main components and assumptions—see Cross and Almberg (2019), Appendices and R code (v3.6.3, R Core Team, 2020) for code and parameter derivations. Table S1 (Appendix S1) specifies the value and source of each parameter.

### 2.1 CWD‐host model

We focused on the invasion of CWD into the elk and mule deer populations in the GYE. We model a disease‐, sex‐ and age‐structured prey population. First we present the mathematical model components (Equation 1), in brief, with a more detailed description following. Let *x* = 1 or 2 for females and males respectively. Age, *a*, is from 1 to the maximum age category *L* (deer = 10 and elk = 18). Ageing is determined by age‐ and sex‐specific survival rates where, on average, females survive longer than males; in addition, individuals can remain in the last age category given they survive, allowing for a skewed age distribution (Gaillard et al., 2000; Bishop et al., 2009; Monteith et al., 2014; Hoy et al., 2020, MacNulty et al., 2020; Appendix S1, Figure S8). Infected prey, *I*, are further subdivided into *j* = 1, 2, …, 10 disease stages such that later stages are more susceptible to predation and transmit infection at higher rates than do earlier stages. We assume individuals moving out of the 10th infectious disease stage die directly from the disease (*γ*). Let *N*(*t*) be the total abundance of the prey population (Equation 1.1):

(1.1) $$N\left(t\right)=\sum_{a=1}^{L}\sum_{x}^{2}S_{x,a}\left(t\right)+\sum_{a=1}^{L}\sum_{x}^{2}\sum_{j=1}^{10}I_{x,a,j}\left(t\right).$$

Let $\lambda_{t}$ be the force of infection, which we define as (Equation 1.2):

(1.2) $$\lambda_{t}=e^{-\left(\frac{\beta_{\text{early}}\sum_{x}\sum_{a}\sum_{j=1}^{7}I_{x,a,j,t}}{N_{t}^{\theta }}+\frac{\beta_{\text{late}}\sum_{x}\sum_{a}\sum_{j=8}^{10}I_{x,a,j,t}}{N_{t}^{\theta }}\right)}.$$

We control the relationship between annual and monthly time using two additional indices *z* and *v* where: IF mod(*z*, 12) = 11, THEN *z* = 1 else *z* = 0, and IF mod(*v*, 12) = 6, THEN *v* = 1 else *v* = 0, so that hunting mortality, *σz*, occurs only in November and is invariant of infection status (Williams et al., 2002), and individuals get 1 year older in June. Let *R*(*t*, *v*) represent the number of offspring born prior to any mortality or disease transmission, a function of age‐specific fecundity *π* (Equation 1.3):

(1.3) $$R\left(t,v\right)=\frac{\mathrm{\pi v}}{2}\left(\sum_{a=1}^{L}S_{x=1,a,t}+\sum_{a=1}^{L}\sum_{j=1}^{10}I_{x=1,a,j,t}\right).$$

Fecundity is equal for infected and uninfected individuals (Edmunds et al., 2016) but reduced for young females (<2 years old; Bishop et al., 2009; Bender & Hoenes, 2018; Stewart et al., 2005; Wright et al., 2006). We assume events occur in the following order: ageing, reproduction, disease progression, transmission, predation, hunting by humans and natural mortality (μ). Let $\tau \left(P_{t}\right)$ be the predation rate as a function of a fluctuating predator population *P*_*t*_. The monthly number of susceptibles *S* of ages greater than 1 can be written as (Equation 1.4):

(1.4) $$S_{x,a+v,t+1}=\left(\left(1-\mu \right)\left(\left(1-\mathrm{\sigma z}\right)\left(\left(1-\tau_{t}\right)\left(S_{x,a,t}\left(1-\lambda_{t}\right)\right)\right)\right)\right).$$

The number of age 1 susceptibles with an even sex ratio is (Equation 1.5):

(1.5) $$S_{x,a=1,t+1}=\left(\left(1-\mu \right)\left(\left(1-\mathrm{\sigma z}\right)\left(\left(1-\tau_{t}\right)\left(\left(S_{x,a=1,t}+R\left(t,v\right)\right)\left(1-\lambda_{t}\right)\right)\right)\right)\right).$$

Infected individuals are (Equations 1.6 and 1.7):

(1.6) $$I_{x,a+v,j=1,t+1}=\left(\left(1-\mu \right)\left(\left(1-\mathrm{\sigma z}\right)\left(\left(1-\tau_{t}\right)\left(\lambda_{t}S_{x,a,t}\right)\right)\right)\right),$$

(1.7) $$I_{x,a=1,j=2:10,t+1}=\left(\left(1-\mu \right)\left(\left(1-\mathrm{\sigma z}\right)\left(\left(1-\tau_{t}\right)\left(\gamma I_{x,a,j-1,t}\right)\right)\right)\right).$$

Chronic wasting disease can be transmitted directly between individuals and indirectly from the environment to an individual—introducing an environmental component to the model would add multiple unknown parameters to an already complicated model. To keep the model more generalizable, we ignored the environmental component but return to this important issue in Section 4. We considered direct transmission to be frequency dependent (Potapov et al., 2013; Samuel & Storm, 2016) on the assumption that group sizes stay broadly similar even as population sizes change, although this is flexible in the model framework (via parameter *θ*) and may vary with scale (see Section 4). Additionally, by applying frequency‐dependent transmission instead of density dependent, the model is likely conservative in the effects of predators on CWD and host dynamics because predation and other causes of mortality reduce host density. Many potential transmission routes are experimentally supported, but the relative importance of these and how that may change over the course of an epidemic wave is poorly understood (Miller et al., 2004). We assumed transmission rates within each sex category were constant for all age classes (Samuel & Storm, 2016).

We used 10 infectious stages to create a gamma distribution of time until disease‐induced mortality and allowed for transmission rates to increase with time since infection. All individuals entered the infected population at CWD stage 1. If they survived, they progressively moved through the 10 stages at rate ρ; hosts could remain in stage 10 for more than 1 month if they continued to survive, and hosts died when they left the last stage at rate *γ*. Median time from infection to death was approximately 23 months for deer (*ρ* = 0.43) and 34 months for elk (*ρ* = 0.28) based on a gamma distribution (shape = 10 and scale = $\frac{1}{\rho }$). These time to death distributions accorded with experimental trials and field‐based monitoring (Appendix S1, Table S1).

**FIGURE 1:**
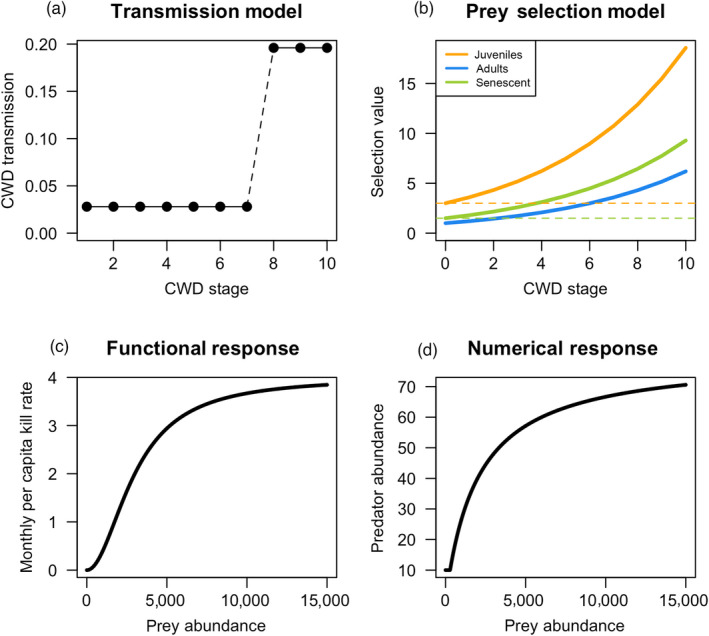
The components of CWD transmission and predation models, as shown for the cougar–deer system. (a) The late‐stage transmission model. (b) Selection values for CWD‐infected individuals increase exponentially as CWD progresses; intersections between the dashed horizontal lines and the selection lines shows the CWD stage beyond which selection for an infected individual from an age class exceeds the selection of an uninfected individual of a different age class (e.g. beyond CWD stage 4, selection of senescent adults is greater than selection of juveniles). (c) Monthly per capita kill rate (*k*) and (d) predator abundance (*P*) as prey abundance increases (*P* range 10–80, *K* = 4, φ = 3,000*,* δ = 2,000)

We selected transmission values (β_*h*_) based on correspondence with observed CWD outbreak dynamics for deer and elk hosts (β_deer_, β_elk_; Appendix S1, Table S2). A range of early and late‐stage transmission values were considered (Appendix S2, Figure S16), and the selected transmission values resulted in logistic growth of CWD prevalence, reaching ~20% by year 20 in deer or year 30 in elk, and maximum prevalence did not surpass 30% by year 30 (Appendix S1, Figure S1). CWD prions tend to accumulate over time within infected individuals, thus we assumed that transmission rates increased in the later stages of infection (stages 8–10) to emulate the observed increase in detected prion shedding in host species (Davenport et al., 2018; Tamgüney et al., 2009; Tennant et al., 2020), and to correspond with the rapid onset of symptoms as the infection progresses (Williams, 2005). Importantly, this transmission model still allowed for transmission from infected animals in the early stages of infection (i.e. asymptomatic) when shedding can be detected at low levels (Davenport et al., 2018; Plummer et al., 2017; Tennant et al., 2020). We assumed transmission rates in late stages (8–10) were five (for elk) and seven (for deer) times higher than in early stages (1–7, β_deer_ *=* 0.028, β_elk_ *=* 0.026), referred to as the late‐stage transmission model (Figure 1a). We developed two other transmission rate models whereby transmission was constant across CWD stages or increased in a linear fashion, which we explored in Appendix S2. Males and females were assumed to have equivalent transmission rates.

All predation and vital rate parameters were derived using empirical data from the GYE or previously published articles from western North America. Elk and mule deer have different vital rates (e.g. deer: higher fecundity; elk: slightly higher survival) and deer have higher rates of CWD transmission and progression than elk.

### 2.2 Selective predation model

Predators remove a prey individual based on its vulnerability (determined by infection status, *j*; Figure 1b) and age class *i* (juvenile: 0–1, adult deer: 2–7, senescent deer: 8–10, adult elk: 2–12 and senescent elk: 13–18 years old); senescence was defined by the reduction in survival and fecundity at age approximately 13 for elk and 8 in deer (Bender & Hoenes, 2018; Bishop et al., 2009; Hoy et al., 2020; MacNulty et al., 2020). Age class ranges are an informed choice that can be adjusted for different species and research questions. Prey selection is the proportion of class *ij* in the predator populations' diet, scaled by its abundance (Equation 3; Johnson, 1980). Predators have a baseline selection for uninfected prey in each age class (wolves: juveniles >> senescent > adults; cougars: juveniles >> senescent ~ adult, Figure 1b *y*‐intercepts). Prey selection increases as the infection progresses; we used an exponential form whereby vulnerability accelerates so that early infections are similarly selected as uninfected sex and age classes (Figure 1b; see Appendix S2 for alternative transmission model forms). Vulnerability is implemented as a multiplicative constant on selection for class *ij*. Because the abundance of each *ij* class varies, we standardized this value for easier interpretation; for instance, a prey selection value of 2 means that the given age and infection stage is killed at twice the rate of some baseline age and infection class if they are equally abundant.

All predator‐related equations (Equations 2–5) are predator specific but not subscripted by predator species *p* for simplicity. For the equation below (Equation 2), *b* is the baseline selection for an uninfected individual of age class *i*, and *r* is the exponential rate of increase in selection as infection progresses (i.e. vulnerability increases). We assume *r* is equal across cougars and wolves in our simulations. Selection prior to standardization, *s*_*ij*_, is:

(2) $$s_{\mathrm{ij}}=b_{i}{\left(1+r\right)}^{j}.$$

We considered wolves to have a wider range in abundance, and cougars to have higher per capita kill rates. All predation parameters were derived using empirical data collected in the northern GYE (Appendix S1).

Baseline selection by age class was determined using selection values standardized such that uninfected adults (*b*_*i* = adult, *j* = 0_) = 1. Selection values were: *b*_*i* = juvenile, *j* = 0, *p* = wolf_ = 3, *b*_*i* = senescent, *j* = 0, *p* = wolf_ *=* 2, *b*_*i* = juvenile, *j* = 0, *p* = cougar_ = 3 and *b*_*i* = senescent, *j* = 0, *p* = cougar_ *=* 1.5 (Appendix S1, Table S3, Figure S3). We used the general form in Equation 2 to calculate the proportion of age *i* and infection stage *j* in the predator population's diet (*f*), and *A* is its abundance. *A* and *f* varied each month, whereas selection values (*s*_*ij*_) were constant throughout the simulation:

(3) $$f_{\mathrm{ij}}=\frac{s_{\mathrm{ij}}A_{\mathrm{ij}}}{\sum s_{\mathrm{ij}}A_{\mathrm{ij}}}.$$

We used a Michaelis–Menten Type III functional response (Bolker, 2008) to model per capita kill rate of each predator species (*k*, Equation 4, Figure 1c) as a function of prey abundance (*N*), maximum per capita monthly kill rate (*K*) and the prey abundance at which *k* is half‐maximal (i.e. inflection point φ; *k = K*/2). We multiplied *k* by predator abundance (*P*) each month to calculate the total number of prey killed by predators (*P* × *k*). *P* varied annually and *N* monthly, while *K* and φ were constant. Thus, *k* was recalculated each month using updated values of prey abundance:

(4) $$k=\frac{K\times N^{2}}{\phi^{2}+N^{2}}.$$

To calculate the number of prey that were removed per class *ij*, we multiplied *k* (Equation 4) by the proportion of class *ij* in the predator's diet (*f*_*ij*_, Equation 3). These prey numbers were then subtracted from the appropriate age and infection class of the prey population. For simplicity, we assume that *K* is the same for male and female prey.

Predator abundance was calculated using a Michaelis–Menten Type II numerical response (Figure 1d), which was determined by the maximum bounds on predator abundance (i.e. max(*P*)), the number of prey (*N*, total prey abundance in May) and the prey abundance at which predator abundance is half‐maximal (i.e. inflection point δ):

(5) $$P_{t+1}=\left(\frac{\max\left(P_{t}\right)\times N}{\delta +N}\right).$$

While Equation 5 allows predator abundance to fall to zero, we set a minimum bound, which is more realistic. Specifically, when *P* the next year (Equation 5) < min(*P*), *P* was set to min(*P*) for the subsequent simulation.

Empirical support for the Type II and Type III numerical and functional responses in Yellowstone wolves is equivocal (Metz, Smith, et al., 2020). To address this, we examined the sensitivity of model outputs to different combinations of Type II and Type III numerical and functional responses (Appendix S2). In general, the Type II numerical and Type III functional responses predicted values of predator/prey abundance and CWD prevalence that were intermediate to those predicted by other combinations of numerical and functional responses. Additionally, over plausible ranges of φ and δ, predator and prey abundances and CWD prevalence were similar. Therefore, we believe our model choice is reasonable and robust; we applied the same models to cougars as no empirical validation exists and these values are reasonable.

### 2.3 Simulations and initialization

We initialized models at stable stage distribution (popbio r package, Stubben & Milligan, 2007). We introduced predators and CWD and ran the simulation for another 20–40 years. CWD was introduced at a low prevalence in calves/fawns (1%), yearlings (3%) and adults (4%), all in CWD stage 1. Following predator and CWD introduction, prey age distribution fluctuated naturally based on the selected parameter values and interactions with predators and disease—prey age distributions well‐matched empirical age distributions (Appendix S1).

## 3 RESULTS

**FIGURE 2:**
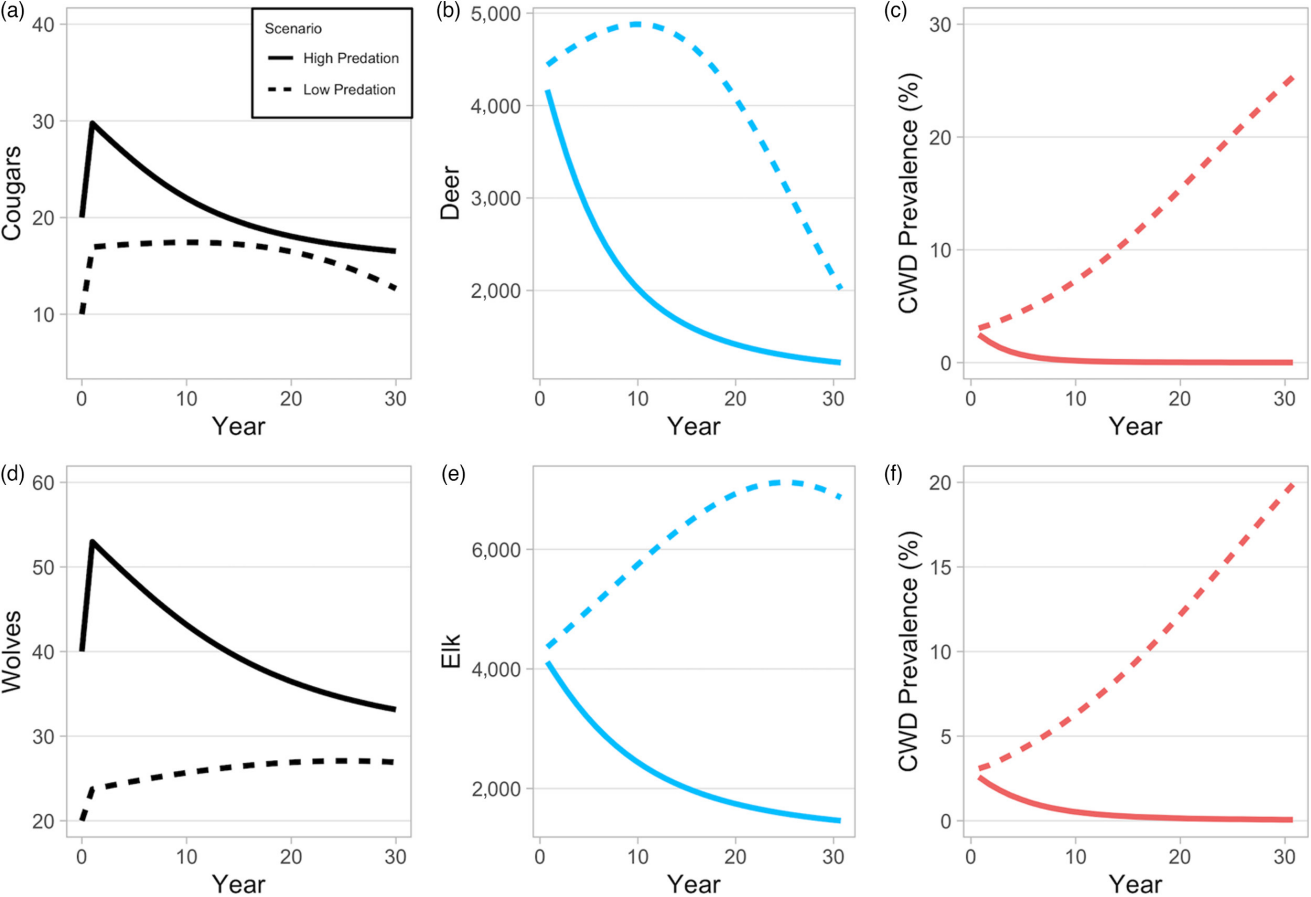
(a, d) Predator abundance, (b, e) prey abundance and (c, f) CWD prevalence under two scenarios: low predation (dashed lines; *r =* 0.1, *K*_wolf_ *=* 0.2, *K*_cougar_ *=* 1) and high predation (solid lines; *r =* 0.35, *K*_wolf_ = 1, *K*_cougar_ = 3) through time

Model results were consistent with empirical data on CWD growth observed in North America (Appendix S1, Figure S1, Table S2) and predation data from the GYE (Appendix S1, Figures S7–S11). In simulations without CWD, wolf kill rates, prey composition and abundance are consistent with empirical data as is elk demography, predation pressure, CWD prevalence abundance and per cent mortality due to predation. Under low predation, CWD increases slowly over the first 5 years following introduction, then consistently increases until year 30 (Figure 2c,f). Mule deer abundance eventually declines under low predation pressure because CWD‐induced mortality rates exceed population growth rates (Figure 2b). The same trend is observed for the elk population, but on a longer time‐scale (Figure 2e). Under most scenarios, there is a sizeable reduction (often nearly 50%), then stabilization, in prey abundance following predator introduction—this trend was also observed in Yellowstone National Park following wolf reintroduction in 1995–1996, driven by a combination of predation, hunting and natural mortality unrelated to predation (MacNulty et al., 2020). In certain high and low predation pressure scenarios, the prey population declines to nearly equivalent levels at year 30 following the introduction of predators, but CWD is substantially reduced or eliminated under high predation pressure (Appendix S2, Figure S13).

Predators with plausible kill rates, selection and abundance can reduce CWD in prey populations. Three parameters describing predation habits are particularly important in reducing CWD outbreaks: (a) adult prey selection (i.e. *b*_*i* = adult, *j* = 10_:*b*_*i* = juvenile, *j* = 0_), (b) selection of infected prey (*s*_*ij*_, function of *r*) and (c) maximum per capita kill rate (*K*).

**FIGURE 3:**
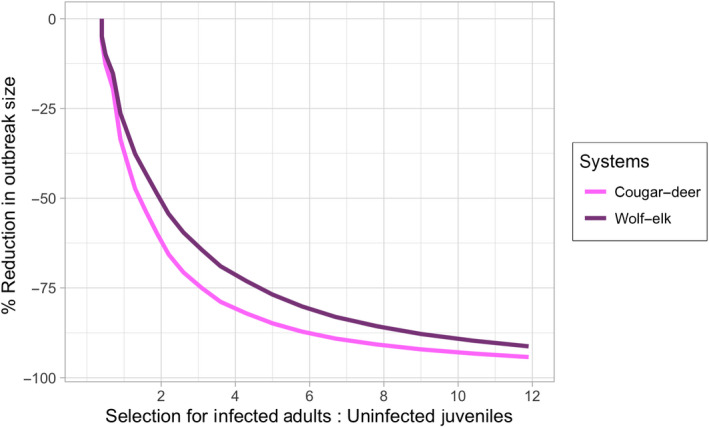
The per cent reduction in outbreak size (cumulative number of CWD‐induced mortalities over a 20‐year simulation) as selection on infected young adults increases compared to uninfected juveniles, predators' selected prey (*b*_*i* = adult, *j* = 10_/*b*_*i* = juvenile, *j* = 0_); for example, a value of 10 means terminally infected young adults (*j =* 10) are selected at 10 times the rate of uninfected juveniles (*r* = 0.01–0.5, *P*_cougar_ = 30, *P*_wolf_ = 50)

**FIGURE 4:**
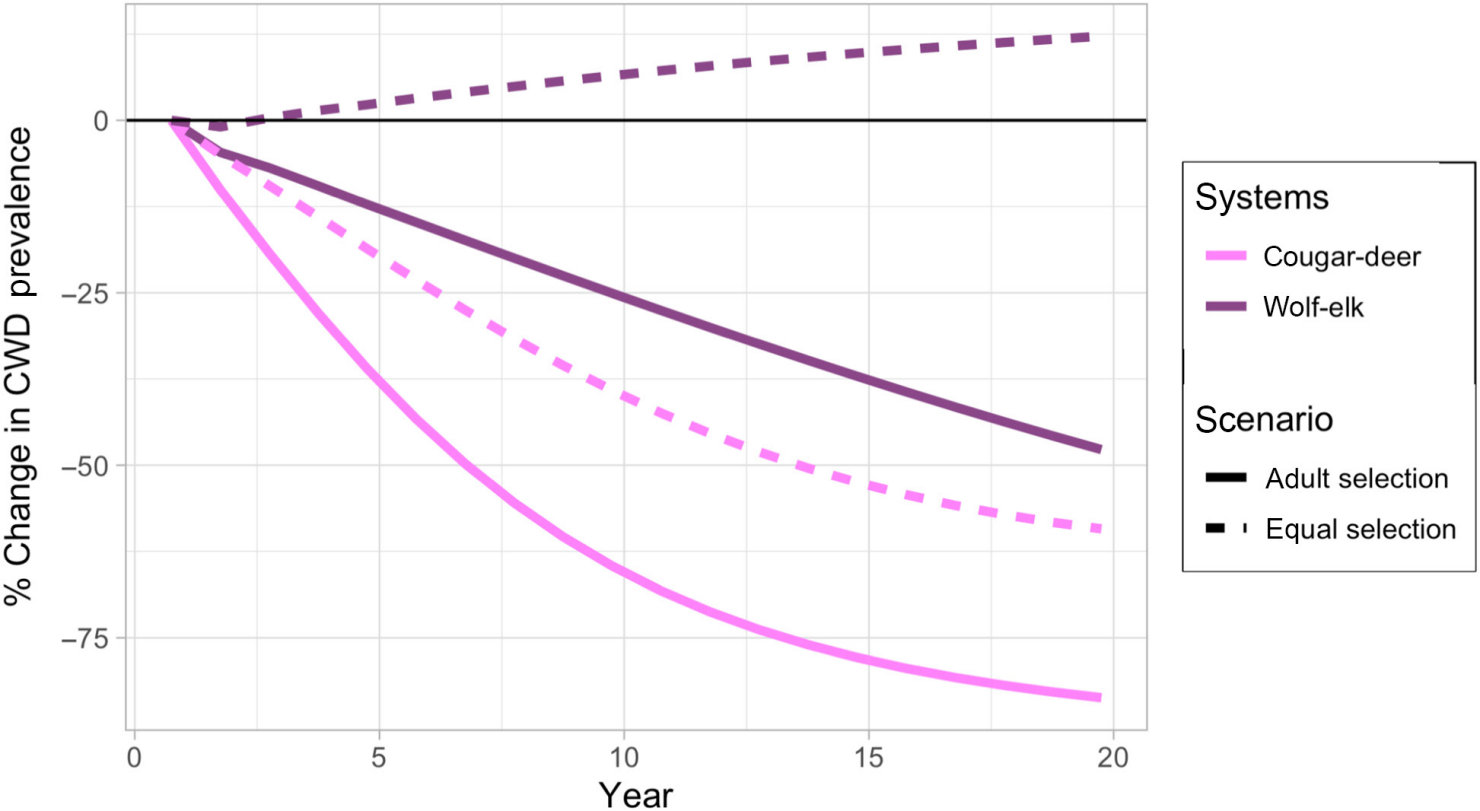
The per cent change in CWD prevalence over a 20‐year simulation when all age classes are selected at the same rate (*b*_*i* = juvenile_ = *b*_*i* = adult_ = *b*_*i* = senescent_, dashed lines) or adults are selected at a higher rate (*b*_*i* = juvenile_ = 1, *b*_*i* = adult_ = 10, *b*_*i* = senescent_ = 5, solid lines). Predation pressure by cougars (pink) and wolves (purple) was moderate (*r* = 0.2, *K*_cougar_ = 2, *K*_wolf_ = 1, *P*_cougar_ *=* 30, *P*_wolf_ *=* 50)

**FIGURE 5:**
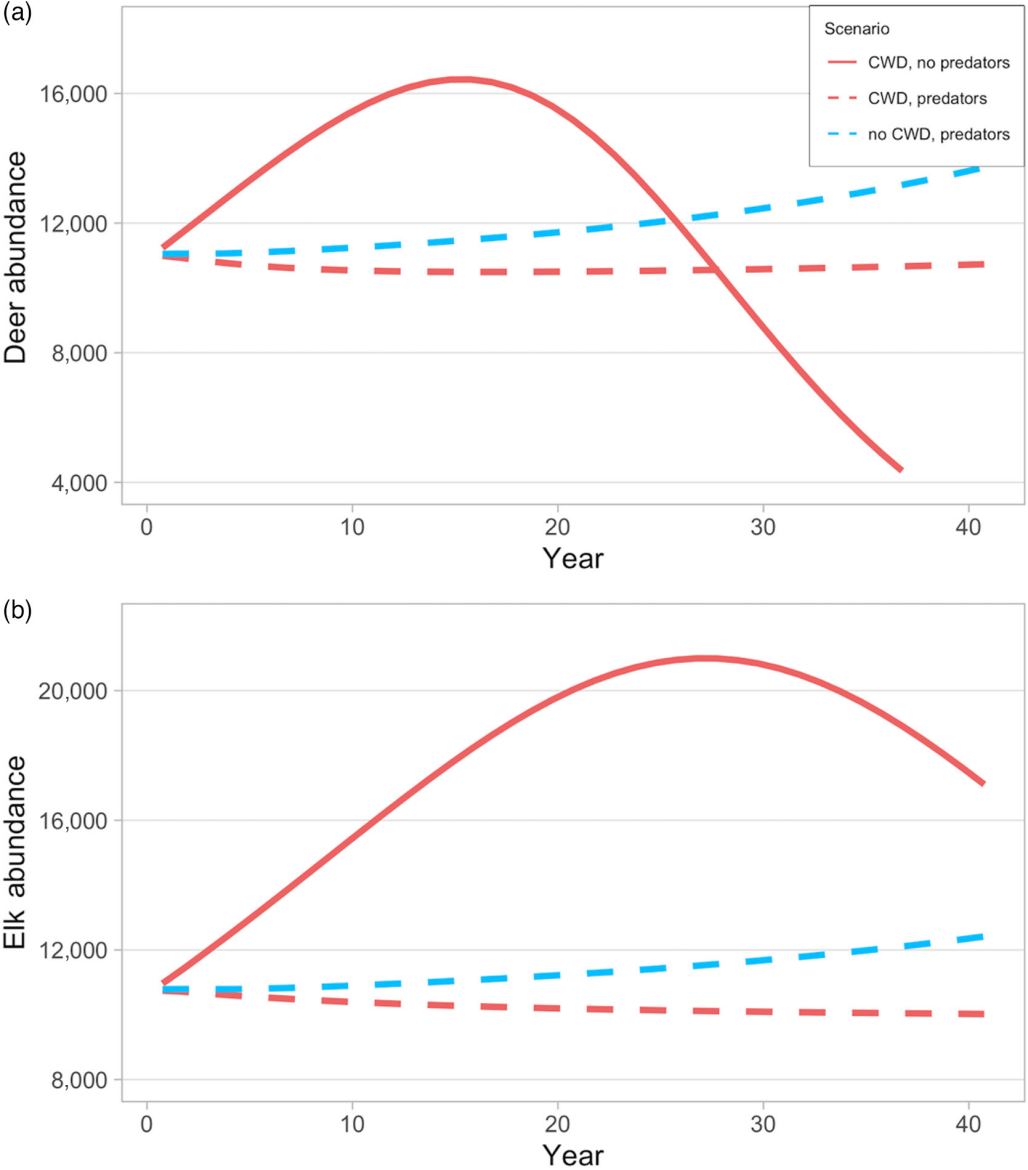
(a) Deer and (b) elk abundance when CWD is present (red) or absent (blue), and predators are present (dashed lines) or absent (solid line) (*K*_cougar_ = 1.8, *P*_cougar_ ≈ 40, *K*_wolf_ = 0.43, *P*_wolf_ ≈ 130, *r* = 0.45). In the ‘CWD and predators’ scenario, CWD prevalence falls to <3% by year 20

1. Outbreaks are reduced the most when severely infected adults (*j* = 8–10) are selected at a higher rate than uninfected juveniles (Figures 3 and 4; Appendix S2, Figure S19E,F). However, host abundance declines when young adults (i.e. prior to senescence) are highly preferred since they are responsible for nearly all reproduction.
2. The level that predators selectively remove infected prey, in comparison to uninfected prey, has a large effect on CWD and prey dynamics. Increasing selection of infected individuals is the most effective way to reduce CWD prevalence and maintain prey abundance with relatively fewer predators. Importantly, a highly selective predator removes CWD and maintains approximately the same abundance as the initial prey population without CWD (Figure 5 dashed red versus dashed blue lines).
3. CWD prevalence and prey abundance are sensitive to predator abundance and kill rate (Appendix S2, Figures S17 and S19C,D). Large predator populations with low kill rates and small predator populations with high kill rates reduce CWD prevalence. However, there is a trade‐off such that higher kill rates result in lower prey abundance with low prevalence (Figure 2). In our simulations, CWD‐induced mortality rate is lower than the predator‐induced mortality rate, so prey abundance could actually be larger, although more infected, when kill rate is low.

When we increase predator pressure to levels sufficient to control the CWD outbreak (≤5% maximum prevalence), predator and prey populations stabilize at plausible levels for single predator–prey systems, and predators are able to control CWD for decades (Figures 2c,f and 5). CWD eradication requires moderate maximum per capita monthly kill rates (*K* ≳ 1.5), strong selection for terminally infected hosts (*r* ≳ 0.3, or >10× greater than uninfected individuals in the same age class) and high predator abundance (≳40/1,000 km^2^). Importantly, sensitivity analyses suggest that changes in individual predation parameters—within a reasonable range—may not be sufficient to eradicate CWD; multiple predation responses acting in unison are more effective to control CWD invasion (e.g. increase *r* and *K* simultaneously; Appendix S2).

Model results indicate that cougar–deer–CWD systems may be more ‘boom and bust’ than wolf–elk–CWD systems. For example, CWD prevalence increases more rapidly in deer populations, but prevalence also declines more quickly than wolf–elk systems (Figures 2 and 4). Additionally, cougars can delay the accumulation of symptomatic deer with fewer predators compared to a wolf–elk system (Appendix S3, Figure S24).

## 4 DISCUSSION

Many authors have proposed predation as a potential biological control mechanism to reduce CWD prevalence in cervid populations and disease in general (DeVivo et al., 2017; Krumm et al., 2010; Miller et al., 2008; Monello et al., 2014; Mysterud & Edmunds, 2019). We developed a host‐predator model that includes host age structure and age‐specific disease and predation patterns to examine predator cleansing effects. Our model results indicate that the interaction between prey selection and disease distribution across host age classes is critical to the effectiveness of predators acting as sanitizing agents in large mammal systems. We expect predators to have the largest dampening effects on disease invasion when the predator displays strong selectivity for severely infected prey, moderate kill rates and at least a moderate abundance. In the GYE, this is approximately a kill rate of at least 1.5 prey/predator/month, >10× greater selection for terminally infected than uninfected prey, and predator density of at least 40/1,000 km^2^, which corresponds well with our derived and observed empirical parameters (Appendix S1, Table S1; Smith et al., 2004). Below we expand on the mechanisms underlying these findings and avenues for the future work.

CWD infection becomes more likely as hosts age, and in endemic settings, young to prime‐aged adults are most likely to test positive (Miller & Conner, 2005; Monello et al., 2014). Mechanistically, removal of adult prey is important because adults are responsible for most transmission events (a combination of abundance and the chronic nature of infection). However, wolves and cougars tend to select juvenile and senescent prey (Hoy et al., 2021; MacNulty et al., 2020; Ruth et al., 2019; Appendix S1), but will reduce CWD more quickly if selection for infected adults surpasses that of juveniles. Our model suggests that selection for severely infected adults needs to be three times higher than selection for juveniles (when juvenile selection is unchanged following CWD invasion) in order to reduce CWD prevalence by approximately 50% in elk and 75% in deer (Figure 3). Understanding predator selection for infected prey is critical to predicting the effects of predators and should be prioritized in monitoring programs.

Kill rate also played an important role in the predator effect on CWD. We posit that kill rate could be the most sensitive response by predators, particularly for wolves, who can dramatically increase their kill rate with readily available prey (Mech et al., 2001). Even highly regulated predator populations can remove CWD and maintain prey abundance under potentially plausible conditions (Figure 5). However, maintaining predator densities high enough to reduce CWD is not only dependent on prey productivity but also on human tolerance. In addition, the interaction between kill rate and selection for infected prey produces a range of results from relatively larger prey populations with higher disease prevalence, to relatively smaller prey populations with low disease prevalence or no disease (Appendix S2, Figures S17 and S19). Therefore, we caution against the interpretation that larger predator populations (functionally equivalent to higher kill rates) are more likely to remove a chronic infection from a prey population. It will be important to examine CWD dynamics in areas with varying predator pressure, harvest rates and prey densities as CWD spreads.

More generally, our goal was to better understand when we might expect to observe relatively strong or weak predator cleansing effects on a prey population. Our results indicate that cleansing effects are amplified when, within the same age class, there is simultaneous selection by predators and aggregation of infection. Conversely, when selection by predators and infection are mismatched, predation will not be able to substantially reduce transmission rates. Some parasites alter host behaviour making them more vulnerable to predation—when the predator is the definitive host, this process can allow for parasite persistence; for example, *Toxoplasma gondii*‐infected hyenas *Crocuta crocuta* have higher mortality rates from African lion *Panthera leo* (definitive host) attacks (Gering et al., 2021). When the predator is not the definitive host, increased vulnerability to predation is a dead end for the parasite and may reduce population prevalence. Predators that are size selective, such as cougars, might exhibit more predictable effects on disease dynamics because their predation strategy is less flexible, while opportunistic predators such as wolves may be more likely to kill hosts with severe disease when they are vulnerable and weak. We examined interactions among prey vulnerability, selection by predators and prey age, but other potentially important interactions should be examined in the future (e.g. decrease in environmental infectious materials through predator digestion, multi‐host dynamics).

Finally, our results have potentially important implications for the monitoring and management of predators and prey. We suggest approaching monitoring predator–prey–pathogen systems by increasing monitoring of the prey population, which is more abundant than predators and often logistically easier to capture and monitor. For example, monitoring prey survival rates and causes of death with respect to disease status can provide insights about predator selection (Krumm et al., 2010; Miller et al., 2008). Still, this can be expensive and produce small sample sizes, especially when incorporating subsets of the prey population such as age class (i.e. one dead ungulate = one sample in one demographic class), making it challenging to detect changes in the short term (Viljugrein et al., 2019). A complementary approach to increased sampling is the development of indicators of CWD in cervid populations that reveal relationships with predators. For instance, our model demonstrates that changes in the ratio of adult prey killed relative to juveniles can suggest a change in the vulnerability of adults, potentially due to disease—this could be enhanced by collecting prey information such as body condition and disease status of ante‐ and post‐mortem prey. If possible, increasing monitoring of the predator population, especially predation habits such as kill rates, will augment these prey data. While monitoring predator–prey–pathogen interactions can be complex, pathogen detection methods are improving (e.g. RT‐QuIC for prions (Cheng et al., 2016) and molecular approaches (DeCandia et al., 2018)), and in some settings it might be fruitful to collect biological samples that can be used for testing as it becomes available in order to increase sample sizes, CWD detection capabilities and improve/test new methods (e.g. predator scats and prey scats/tissues can be collected at prey kill sites, foraging locations and live captures; see Appendix S3 for further management discussion).

We simplified model structure in order to keep our findings applicable to other systems and to reduce additional parameter uncertainty. For instance, controlling the accumulation of infectious hosts likely has implications for environmental contamination, and a dynamic environmental reservoir could be incorporated in the future CWD models once there are empirical data for parameterization. Still, predators will probably reduce the accumulation of environmental contamination if they selectively remove hosts faster than a disease kills them, even if temporarily predators pass infectious agents after ingestion (e.g. prions: Nichols et al., 2015). Additionally, CWD transmission has been described as an intermediate between frequency and density dependent (Almberg et al., 2011; reviewed in Ketz et al., 2019). Often this distinction between transmission models depends upon the spatial scale of the model or analysis as well as how the host grouping patterns are associated with changes in population size. We assumed that, at the population level, prey group size, contact rates and transmission rates may be relatively constant as predators reduce the population size of prey. Our frequency‐dependent model likely demonstrates a conservative effect of predators on CWD dynamics. Elk population size in the GYE is uncorrelated with mean group size, but the largest elk groups do get larger with increasing populations and brucellosis has been correlated with elk density (Brennan et al., 2015; Cross et al., 2010; Proffitt et al., 2015). Thus, there is likely some density dependence in elk CWD transmission at the population scale. Large aggregations of elk on supplemental feedgrounds are also likely to increase disease transmission (NAS, 2017). On the other hand, some ungulate populations have displayed a decrease in group sizes as density declines, while the number of groups in the population remains constant (McLellan et al., 2010). Our model framework can accommodate a gradient from frequency‐ to density‐dependent transmission, making it adaptable to many systems and types of exploration. Further, the co‐evolution of pathogen (e.g. CWD strain, Velásquez et al., 2020) and hosts (e.g. PRNP gene, elk: Monello et al., 2017; mule deer: LaCava et al., 2021) expands the application of the model.

Like any model, we made assumptions about the relationships between predators and prey and also about hosts and pathogen infection. Mortality from disease and predation were partially compensatory, but these causes of mortality were additive with respect to natural mortality and harvest. This area should receive future empirical validation; for example, CWD‐infected male mule deer may be more susceptible to harvest (i.e. partially compensatory mortality; Conner et al., 2000). Managers can also control this by, for example, issuing fewer female hunting tags when the population is declining. Given the model complexity, we considered pathogen and predation dynamics to operate the same for male and female prey, which should be further explored via models and empirical validation in the future. For instance, male mule deer tend to have higher observed CWD prevalence than female mule deer (e.g. Miller & Conner, 2005), although the underlying mechanisms are currently unknown. The addition of sex‐specific transmission rates could account for this observation, which may have subsequent impacts on sex‐specific predation rates. Similarly, in our system, wolf predation by demographic class shifts seasonally (Metz, Hebblewhite, et al., 2020), which could influence disease dynamics if infections were additionally aggregated by sex, as mentioned above. Transmission rate greatly increased with time since infection in our model, but we emphasize the need for empirical quantification of CWD infection parameters such as transmission rate and prion shedding rate throughout the disease course. Protected areas where predator populations are largely conserved will serve as an important comparison for understanding the interactions between predators, prey and disease in the coming years to decades.

## 5 CONCLUSIONS

Parasite infections in wildlife hosts and prey selection by predators are often age specific. We examined the potential effects of age‐selective predators on prey populations with imminent CWD invasion in the GYE. Our results suggest that CWD outbreaks are reduced as predators increase their selection for younger adults, which is the age class with the highest disease prevalence, but not necessarily the class that predators typically select. Additionally, predators that were highly selective for infected hosts promoted healthy prey populations while maintaining prey abundance under certain circumstances. Our results support the hypothesis that the predator cleansing effect will be more efficient in systems where disease and predation target the same prey age groups, and this can be empirically evaluated in the coming years as CWD and predator distributions increasingly overlap.

## CONFLICT OF INTEREST

The authors have no conflict of interest to declare.

## AUTHORS' CONTRIBUTIONS

E.E.B. constructed the predation models, ran all simulations and wrote the first draft of the manuscript; P.C.C. developed the CWD‐host Leslie matrix model. All authors developed the hypotheses and contributed substantially to manuscript revisions.

## Supporting information

Supplementary Material

Supplementary Material

Supplementary Material

## Data Availability

A vignette, model code and some plotting features are available via USGS as an addition to Cross and Almberg (2019), DOI: https://doi.org/10.5066/P9QZTTLY. GitHub site: https://code.usgs.gov/usgs/norock/cross_p/cwdsims.
