# Supplementary material for: Examination of the interaction between age‐specific predation and chronic disease in the Greater Yellowstone Ecosystem
Source: J Anim Ecol. 2022 Jan 27;91(7):1373–84. doi: 10.1111/1365-2656.13661 (PMC9912199; doi:10.1111/1365-2656.13661)
Supplement: Supplementary file 1 — Supplementary Material [file JANE-91-1373-s002.pdf]

Supporting Information Appendix 1: Model Parameterization and Validation

Examination of the interaction between age-specific predation and chronic disease in the Greater Yellowstone Ecosystem

Ellen E. Brandell, Paul C. Cross, Douglas W. Smith, Will Rogers, Nathan L. Galloway, Daniel MacNulty, Daniel R. Stahler, John Treanor, Peter J. Hudson

Table S1. Parameter symbols, descriptions, values, and references.

symbol	parameter	estimate	calculation / rationale	reference
L	Elk number of age categories<br><br>Deer number of age categories	18<br><br>10	Note that the model allows individuals to remain in the final age class based on survival rates, so lifespan can slightly exceed the last age class.	Yellowstone Wolf Project, Wright et al. 2006, Hoy et al. 2020<br><br>Bishop et al. 2005, 2009
$\mu_{a=1,\ h=elk}$<br><br>$\mu_{a=1,\ h=deer}$	Calf/fawn survival	0.7<br><br>0.5		Barber-Meyer et al. 2008, Eacker et al. 2016, Hoy et al. 2020<br><br>White et al. 1987, Bishop et al. 2009, Forrester and Wittmer 2013, Monteith et al. 2014
$\mu_{a=2,\ h=elk}$<br><br>$\mu_{a=2,\ h=deer}$	Yearling survival	0.9<br><br>0.8		Lubow and Smith 2004, Wright et al. 2006, Hoy et al. 2020<br><br>White et al. 1987
$\mu_{a=3:L,\ h=elk}$<br><br>$\mu_{a=3:L,\ h=deer}$	Adult survival	Female: 0.9<br>Male: 0.8<br><br>Female: 0.9<br>Male: 0.85		Evans et al. 2006, Wright et al. 2006, Hoy et al. 2020<br><br>White et al. 1987, Bishop et al. 2005, 2009, Forrester and Wittmer 2013, Bender and Hoenes 2018
$\pi_{a=2,\ h=elk}$<br><br>$\pi_{a=2,\ h=deer}$	Yearling fecundity	0.4<br><br>0.4		Stewart et al. 2005, Wright et al. 2006<br><br>Bishop et al. 2009, Monteith et al. 2014, Bender and Hoenes 2018
$\pi_{a=3:L,\ h=elk}$<br><br>$\pi_{a=3:L,\ h=deer}$	Adult fecundity	0.9<br><br>1.2		Stewart et al. 2005, Wright et al. 2006, Hoy et al. 2020<br><br>Bishop et al. 2009, Forrester and Wittmer 2013, Monteith et al. 2014, Bender and Hoenes 2018
$\sigma_{a=1,\ h=elk/deer}$	Harvest rate: calves/fawns	0.01		
$\sigma_{x=F,\ a=2,\ h=elk}$	Harvest rate: yearling female	0.03<br><br>0.02		

$\sigma_{x=F,\ a=2,\ h=deer}$				
$\sigma_{x=M,\ a=2,\ h=elk}$<br><br>$\sigma_{x=M,\ a=2,\ h=deer}$	Harvest rate: yearling male	0.03<br><br>0.02		
$\sigma_{x=F,a=3:L,h=elk}$<br><br>$\sigma_{x=F,a=3:L,h=deer}$	Harvest rate: adult female	0.05<br><br>0.04		Evans et al. 2006, Wright et al. 2006<br><br>Mule Deer Working Group 2019
$\sigma_{x=M,a=3:L,h=elk}$<br><br>$\sigma_{x=M,a=3:L,h=deer}$	Harvest rate: adult male	0.1<br><br>0.1		 <br>Mule Deer Working Group 2019
γ	CWD-induced mortality in matrix model notation (Eq. 1)			
τ	Mortality due to predators in matrix model notation (Eq. 1)		Determined by equations 2-5	
Subscript *x*	Host sex: female (*F*), male (*M*)			
Subscript *a*	Host age in years (*a*>*L* is grouped into *L*)			
Subscript *h*	Host species: elk or mule deer			
Subscript *i*	Age class: juvenile, adult, senescent			
Subscript *j*	Infection stage 0-10 where 0 is uninfected and 10 is the final stage before mortality			
Subscript *p*	Predator species: wolf or cougar			
$b_{i=juvenile,\ j=0,\ p}$	Selection for juvenile elk/deer<br>*p=wolf*<br>*p=cougar*	 3<br>3	Adults are considered baseline = 1	Wright et al. 2006, Wilmers et al. 2020, Appendix 1
$b_{i=adult,\ j=0,\ p}$	Selection for adult elk/deer<br>*p=wolf*<br>*p=cougar*	 1<br>1	Adults are considered baseline = 1	Wright et al. 2006, Wilmers et al. 2020, Appendix 1

$b_{i=senescent, j=0, p}$	Selection for senescent elk<br>*p=wolf*<br>*p=cougar*	 2<br>1.5	Adults are considered baseline = 1<br><br>Senescence was defined by the reduction in survival and fecundity that occurs around age 13 for elk and 8 in deer.	Wright et al. 2006, Bender and Hoenes 2018, Hoy et al. 2020, Appendix 1
K<br><br>K_{wolf}<br><br>K_{cougar}	Maximum monthly per capita kill rate<br><br>Wolf-elk<br><br>Cougar-deer	 <br>0.2-2.5<br><br>1-6		Informed by Yellowstone Wolf Project data and Yellowstone Cougar Project data, Evans et al. 2006, Varley and Boyce 2006, Barber-Meyer et al. 2008, Metz et al. 2012, Ruth et al. 2019, Appendix 1
	Relative harvest risk (susceptible host:infected host)	1:1		Williams et al. 2002
s_{ij}<br><br><br><br>r, c	Prey selection by age class (*i*) and infection stage (*j*)<br><br>Functional form (vulnerability)<br><br>Rate of increase in vulnerability using the exponential or linear model, respectively	Calculated within model<br><br>Exponential<br><br>*r*: 0.05-0.5<br>*c*: 0.5-1.5		
	Numerical response<br>Functional response	Type II<br>Type III	Wolves and cougars have the potential to switch prey at low elk densities (but see Tallian et al. 2017).	Metz et al. 2020, Appendix 2
	Sex ratio at birth	50:50		
ϕ<br><br>$\phi_{h=elk}$<br>$\phi_{h=deer}$	Inflection point in kill rate for functional response<br>Wolf-elk<br>Cougar-deer	 <br>3000<br>3000		Informed by Yellowstone Cougar Project data, Metz et al. 2020
δ<br><br>$\delta_{h=elk}$<br>$\delta_{h=deer}$	Inflection point in population growth for numerical response<br>Wolf-elk<br>Cougar-deer	 <br>2000<br>2000		Informed by Yellowstone Cougar Project data, Metz et al. 2020
β_h<br><br><br><br>M_h	Baseline transmission rate by host species: deer or elk<br><br><br>Increase in transmission in late-stage versus early infections by	Deer: 0.028<br>Elk: 0.026<br><br><br>Deer: 7<br>Elk: 5	See Fig. S1, S16<br><br><br>Based on empirical support, deer transmission > elk.	Miller et al. 2000, 2008, 2020, Miller and Conner 2005, Williams 2005, Joly et al. 2006, Almberg et al. 2011, Storm et al. 2013, Samuel and Storm 2016

	host species: deer or elk			
	CWD transmission functional form (i.e., increase in transmission rate β_h as CWD progresses)	Late-stage, exponential, linear, equal	Hosts have a long asymptomatic phase, followed by a sharp increase in symptoms; this corresponds to higher prion titers and likely reflects prion shedding rates (i.e., transmission).	Williams et al. 2002, Fox et al. 2006, Tamgüney et al. 2009, Davenport et al. 2018
	Initial CWD prevalence fawn/calf deer or elk	0.01		Informed by Miller et al. 2000
	Initial CWD prevalence yearling deer or elk	0.03		Informed by Miller et al. 2000
	Initial CWD prevalence adult and senescent deer or elk	0.04		Informed by Miller et al. 2000
θ	Frequency / density dependent transmission	1	Entirely frequency dependent.	Williams et al. 2002, Potapov et al. 2013, Samuel and Storm 2016
ρ	Probability of progression to next CWD stage (1-10)	Deer: 0.43<br>Elk. 0.28	Based on average time to death from infection.<br>Deer: ~23 months<br>Elk: ~34 months	Williams et al. 2002, Williams 2005, Fox et al. 2006, Robinson et al. 2012, Plummer et al. 2017, Moore et al. 2018, Mysterud and Edmunds 2019
P<br><br>P_{wolf}<br>P_{cougar}	Predator abundance	 <br>~20-150<br>~10-80	Predator ranges considered, slightly above and below actual population counts.<br><br>Initial varies by simulation; subsequent size calculated within the model.	Informed by Yellowstone Wolf Project data and Yellowstone Cougar Project data, Varley and Boyce 2006
N	Prey (host) population size / abundance		Initial varies by simulation, often 4000; subsequent size calculated within the model.	
f_{ij}	Proportion of each age class (i) and infection stage (j) in predator population's diet		Calculated within the model.	
A_{ij}	Abundance of each prey age class (i) and infection stage (j)		Calculated within the model.	

Parameterizing transmission rates

Based on the sharp increase/appearance of clinical symptoms in late-stage infections that correspond with high prion titers (see Table S1 for sources), we wanted the increase in β in later CWD stages to be at least three-times larger than early stages. M is considered the 'scaling factor', or the magnitude of increase from early to late-stage transmission: late-stage β = early stage β*M. We explored a range of early and late-stage β values and selected β values based on how well they corresponded to empirical observations (Table S2, Fig. S1, Appendix 2 Fig. S16). Empirical elk data were lacking, but in general, elk were considered to have lower prevalence (Miller et al. 2000). Table S2 informed our parameter selection, which resulted in logistic growth of CWD prevalence, reaching ~20% by year 20 in deer or year 30 in elk, and maximum prevalence did not surpass 30% by year 30 (Fig. S1).

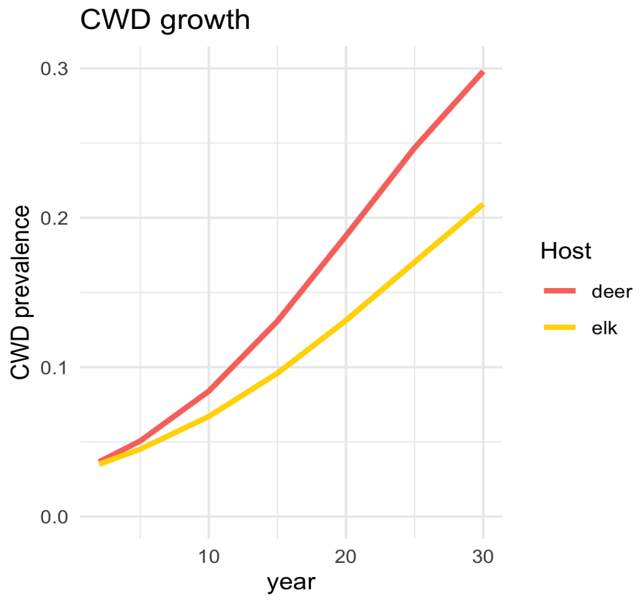

Figure S1. Modeled CWD prevalence over time for deer (red) and elk (gold) hosts. Predators are not included.

Table S2. Deer (mule[m] and white-tailed[w]) CWD prevalence, population or CWD status, and sources that informed our transmission parameters and resulting CWD growth.

Prevalence	Population/CWD status	Source
Initial ≥3%, Peak ≤50% (at 10 years)	Model based on empirical data	Almberg et al. 2011[m]
Male 41%, Female 20%	Endemic	Miller et al. 2008[m]
~10%, never above 20%	Endemic	Miller et al. 2020[m]
~6% (~10% in infected herds)	Endemic	Miller et al. 2000[m/w]
Male ~10%, Female ~5%	Endemic	Miller and Conner 2005[m]
Range used 7-20%	Model based on empirical data from invasion	Storm et al. 2013[w]
All <10%, mostly ~5%	Endemic	Samuel and Storm 2016[w]
All <10%, mostly ~2%	Early invasion	Joly et al. 2006[w]

Selective predation model

Predators often select prey based on age, size, or condition (Kunkel et al. 1999, Husseman et al. 2003, Mech and Barber-Meyer 2015, Ruth et al. 2019, MacNulty et al. 2020). We constructed three selection models that allow for prey selection by age class and CWD stage: linear, equal, and exponential (Fig. S2). For a cursorial predator like a wolf, the exponential model is more realistic, and it is what we used in the main text.

Linear selection: $s_{ijp} = b_{ijp} + j*c$ (Eq. S1)

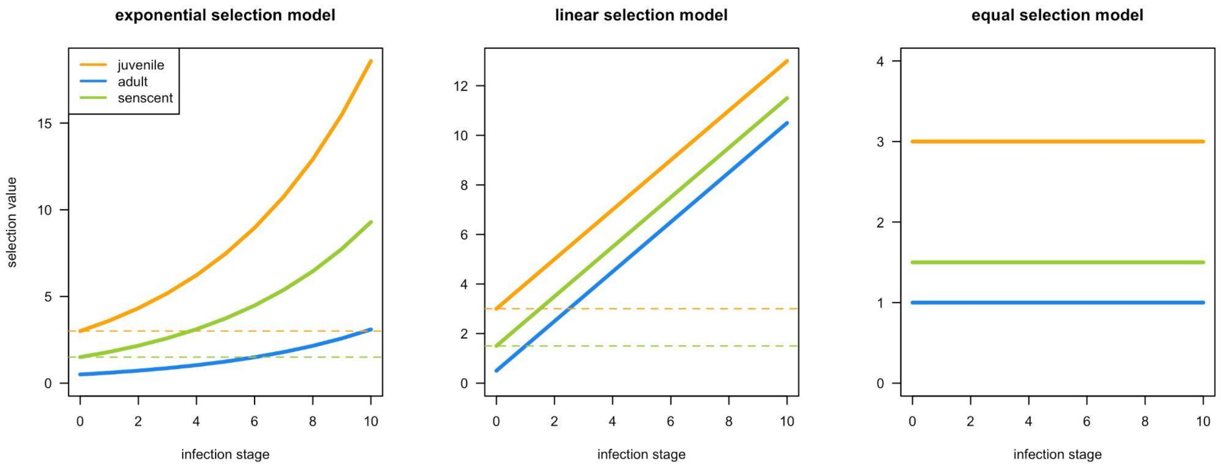

Figure S2. Plot displaying prey selection models with different functional forms: (A) exponential, (B) linear, and (C) equal. Prey selection is a function of CWD stages (j = 0-10) and age class (juveniles = orange, adult = blue, senescent = green; c = 1). Stage 0 are healthy/uninfected individuals and thus the y-intercept is the baseline selection by age class.

Baseline prey selection by age class was derived using a standard selection model (Johnson 1980, Lele et al. 2013): % ij in p diet / % ij in N population. We then standardized selection so that adults are the reference age class ($b_{i=adult,\ j=0}$ = 1), giving us relative measures of selection. We used data from the wolf kills and reconstructed elk population age structure data from the northern Greater Yellowstone Ecosystem to calculate selection (Hoy et al. 2020). We used years with high quality wolf kill composition and elk counts: 1998-2009 (except 2002 and 2006). This gave us a range of plausible selection values (Table S3, Fig. S3). Selection for juveniles and senescent adults were very high in early years, but stabilized from 2004-2009, thus we used $b_{i=juvenile,\ j=0,\ p=wolf} = 3$, $b_{i=senescent,\ j=0,\ p=wolf} = 2$, and $b_{i=adult,\ j=0,\ p=wolf} = 1$.

Table S3. Baseline wolf selection by elk age class in northern Greater Yellowstone Ecosystem, standardized to adults.

**year**	$b_{i=juvenile, j=0}$	$b_{i=senescent, j=0}$	$b_{i=adult, j=0}$
1998	7.24	6.40	1
1999	5.55	6.28	1
2000	4.14	8.35	1
2001	4.30	8.86	1
2003	4.14	5.48	1
2004	2.00	2.82	1
2005	2.43	2.92	1
2007	3.23	2.76	1
2008	2.81	2.22	1
2009	3.67	2.04	1

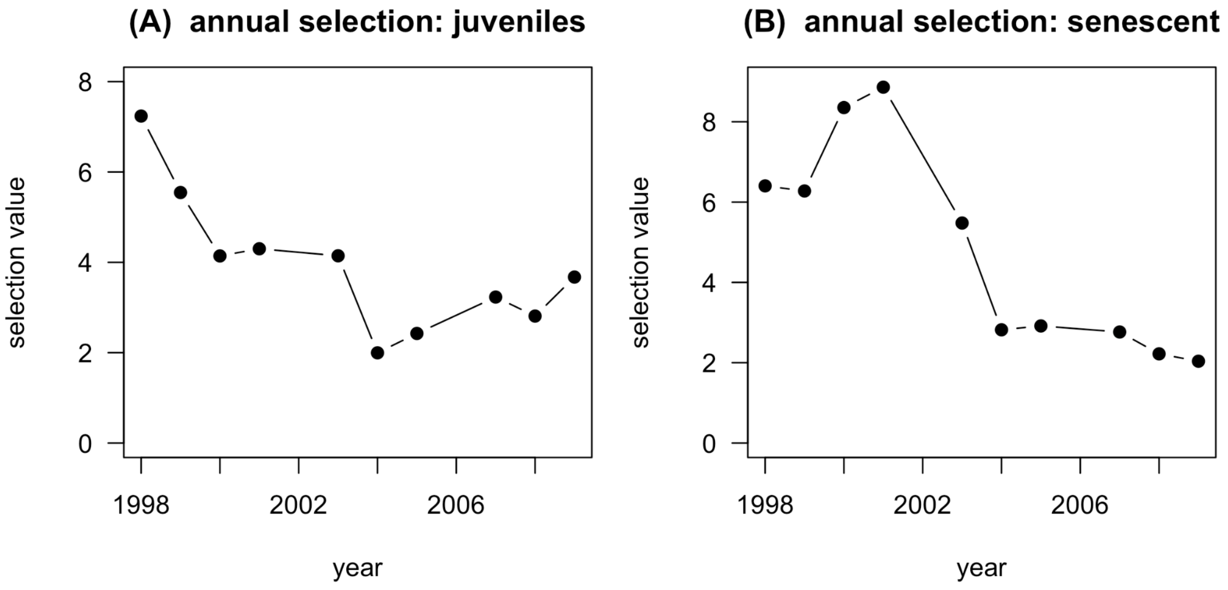

Figure S3. Wolf prey selection through time for (A) juvenile and (B) senescent adult elk with reference to adults ($b_{i=adult, j=0} = 1$); interpretation of selection is the number of individuals in class *ij* that would be killed by wolves compared to adults, given equal abundance.

Mule deer demographic data for Yellowstone included fawn, juvenile, and adult counts from 2016-2019 (Fig. S4). We used cougar kill demographics and mule deer counts from 2018 (Montana hunting district 313, Montana Fish Wildlife & Parks) as well as Yellowstone National Park to calculate selection values. This resulted in $b_{i=juvenile, j=0, p=cougar} = 3$ with $b_{i=adult, j=0, p=cougar} = 1$. Based on cougar hunting habits, we expected senescent adults to be marginally selected over adults, so we set $b_{i=senescent, j=0, p=cougar} = 1.5$ for our cougar-deer simulations as no empirical data were available for this age class.

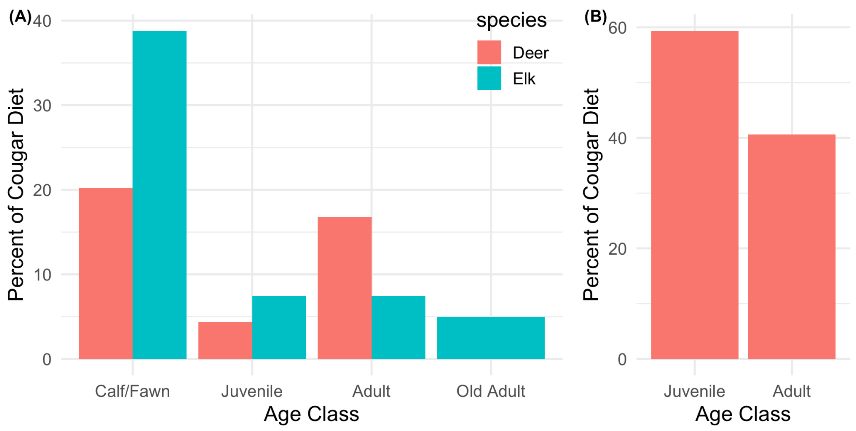

Figure S4. Cougar diet composition from 2016-2019 in northern Yellowstone National Park. (A) Percent of mule deer (red) and elk (teal) in cougars' diet with the highest resolution in age possible given the data. (B) The age composition of mule deer killed by cougars that is relevant to our model calibration – i.e., fawns and juveniles are aggregated into one "juvenile" class, and old adult deer were not able to be classified separately from adult deer.

We calculated the monthly kill rate for wolves and cougars using data from winter. For wolves, we took the average of an intensive 30-day study period – the month of March – from 1998-2010. We had fewer years of data for cougars, so we used multiple months when cougars are tracked in the field (February, March, May, June, mid-Nov–mid-December) from 2016-2019 for our estimates. 94-100% of wolf kills were elk, thus we made the assumption that all kills were elk throughout our calculations. Our parameter space was also informed from (Metz et al. 2012, 2020) – they estimated wolves consume ~0.04 ungulates/wolf/day, or ~1.2 ungulates/wolf/month. Deer comprised about half of cougars' diet (monthly mean = 46%, median = 53%, range 11-68%). We calculated the probable range of monthly kills of the respective host species using the long-term average, minimum, and maximum percentage of kills of that host.

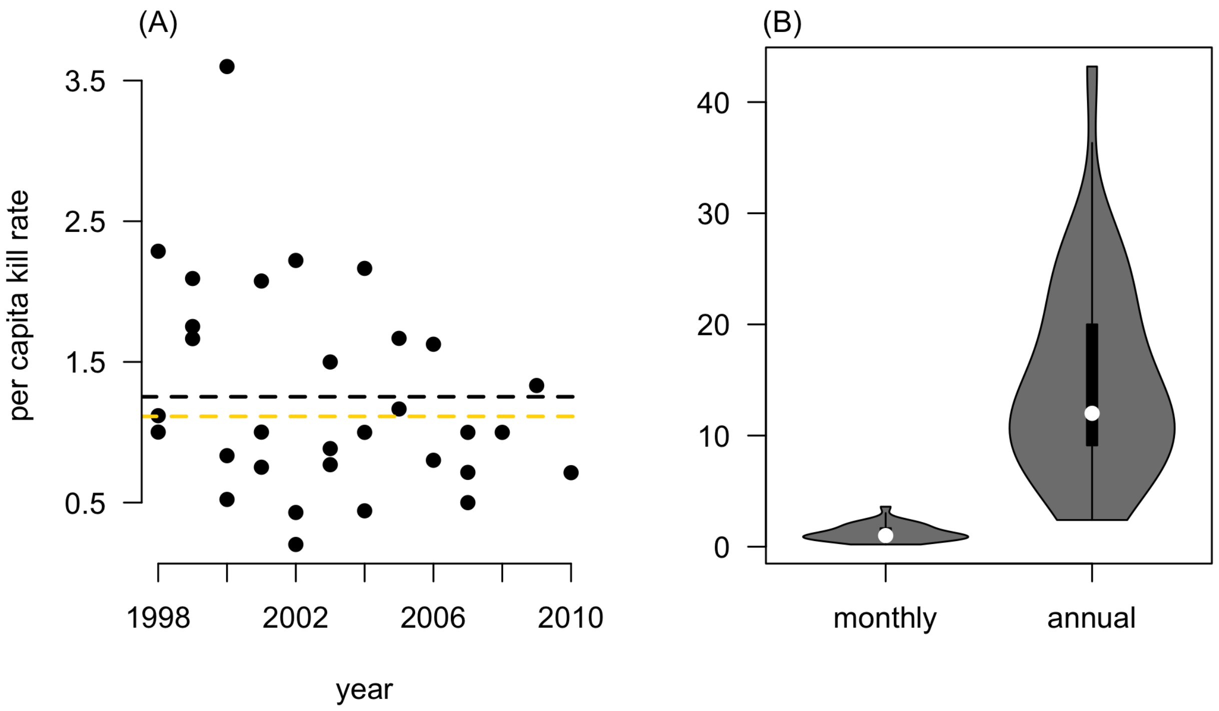

Figure S5. (A) Per capita monthly kill rates (March) for Yellowstone wolves from 1998-2010. Mean kill rate (all detected prey, black dashes) is nearly equal to mean elk kill rate (gold dashes). (B) Distribution of per capita monthly and annual kill rates.

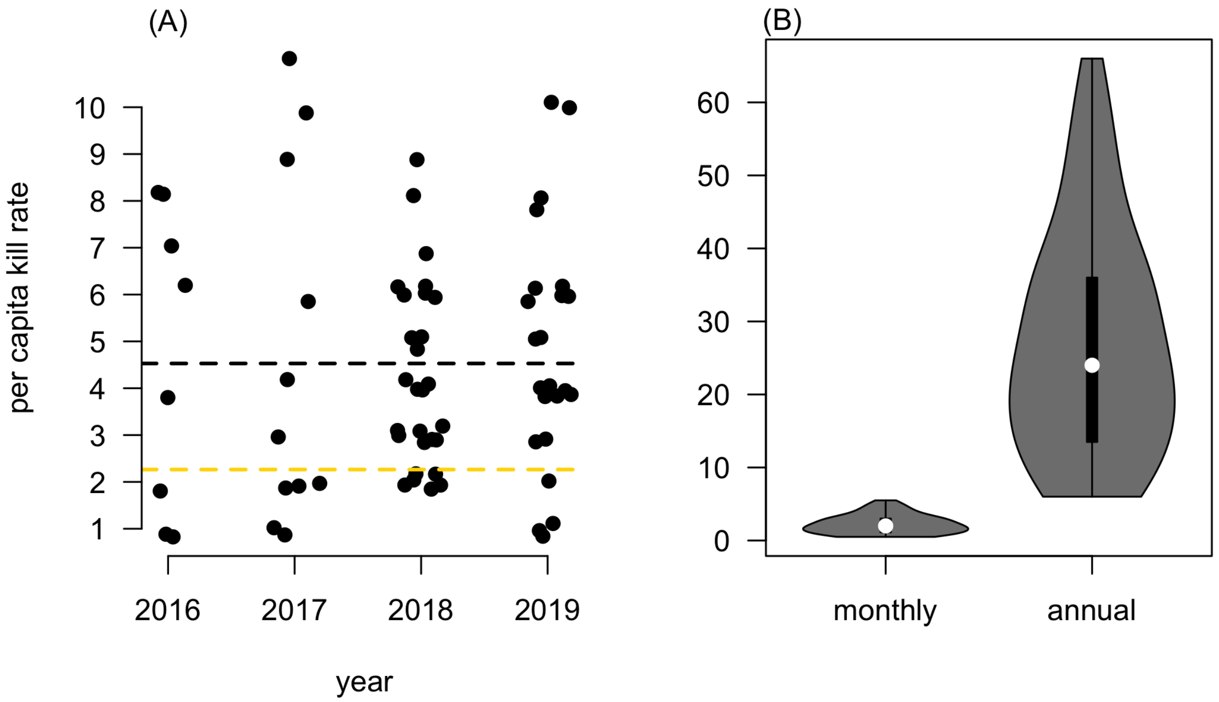

Figure S6. (A) Per capita monthly kill rates for Yellowstone cougars from 2016-2019. Imputed deer kill rate (gold dashes) is half of mean kill rate (all detected prey, black dashes). (B) Distribution of estimated per capita monthly and annual deer kill rates.

To evaluate how well our model matched empirical wolf-elk and cougar-deer data in Yellowstone, we ran a simulation with approximately no CWD (β = 0.01, initial prevalence ≤1%), and plotted outputs with respect to predator-prey relationships and elk demography below. All results are from the same simulation (Fig. S7-S11). Wolf kill rate, and variation in kill rate (±1 standard deviation), from the simulation are within empirical bounds (Fig. S9). We find that elk demography is well matched with empirical data (Fig. S8A, Fig. S10A, Hoy et al. 2020, 2021). More specifically, even though males and females have the same number of age categories in our model, it is unlikely that males live as long as females due to their lower annual survival and higher hunting rates. In Figure S8A, the increase in frequency in the last age category (18) represents individuals who survived until that category or longer, and female elk are more likely to live to these greater ages than males. Using the same age categories as Hoy et al. (2020), the elk population is 48% adults (2-8 years old), 38% juveniles (<2 years old), and 14% senescent (≥9 years old) – this corresponds well with long-term data. Figure S10A shows the proportion of the wolf population's diet of each age class, which also corresponds well with empirical data (Metz et al. 2012). Finally, the proportion of elk mortalities attributed to wolves and humans is plausible (Figure S11A).

Unfortunately, high resolution mule deer demographic data are not available in our system, but the patterns are similar: survival declines with age (and more rapidly for older adults), and females have higher survival rates (i.e., longer average lifespans) than males (Bishop et al. 2005, 2009, Bender and Hoenes, 2018). Therefore, we are confident our model appropriately captures host demography for our modeling purposes. We included relevant plots from the cougar-deer model where we were able to compare with empirical data. Overall, the predation component of our model mimics the Yellowstone ecosystem reasonably well.

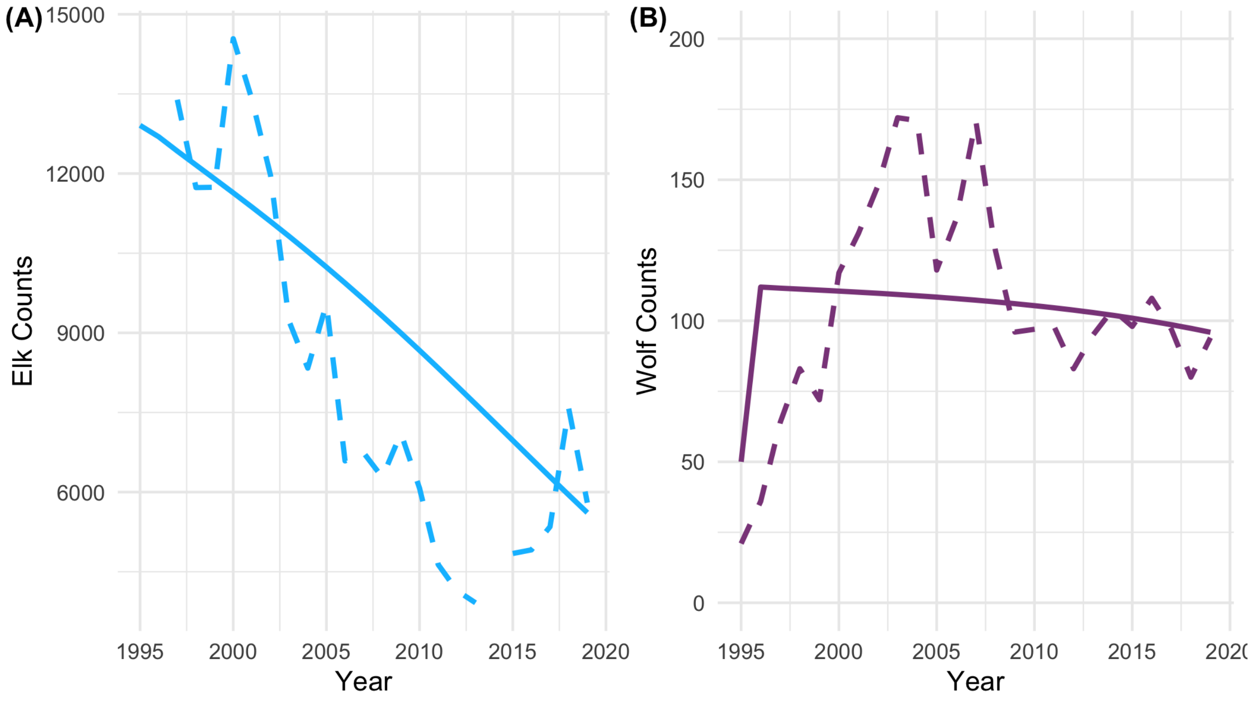

Figure S7. Model output (solid lines) compared with empirical counts (dashes) for the (A) Northern Yellowstone elk population and (B) Yellowstone wolf population.

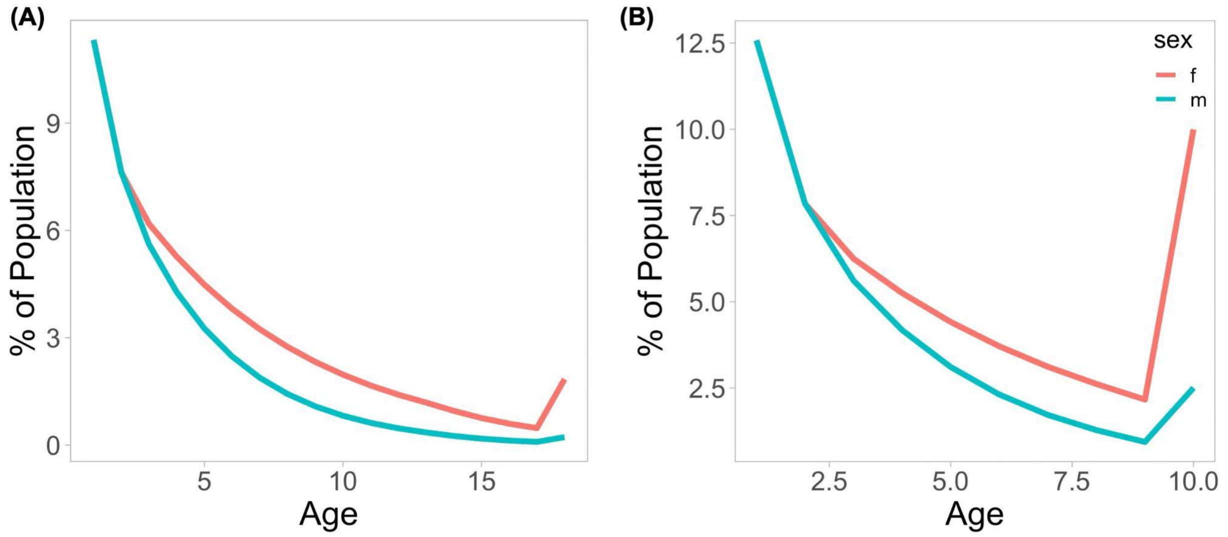

Figure S8. Percent of the (A) elk and (B) mule deer population by age at the end of the 25-year simulation, colored by sex (red f = female, turquoise m = male).

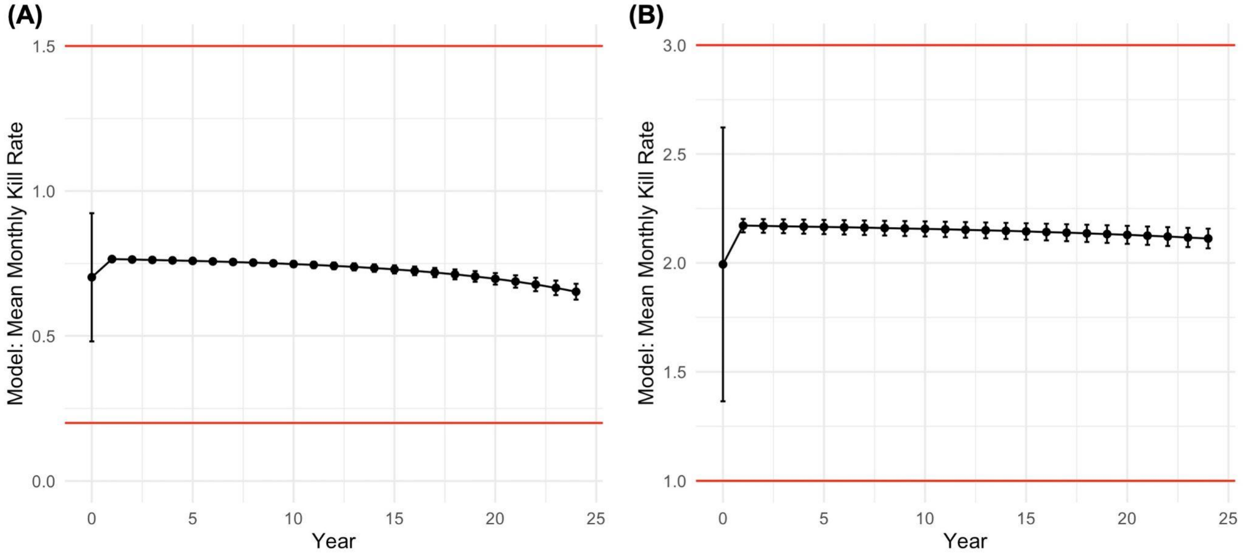

Figure S9. Model output of monthly kill rate for (A) elk (# elk killed/wolf/month) and (B) mule deer (# deer killed/cougar/month) averaged for each year (± 1 SD). The red lines are approximate bounds on monthly kill rate based on empirical data (Fig. S4, Fig. S5, Metz et al. 2020, Yellowstone Cougar Project).

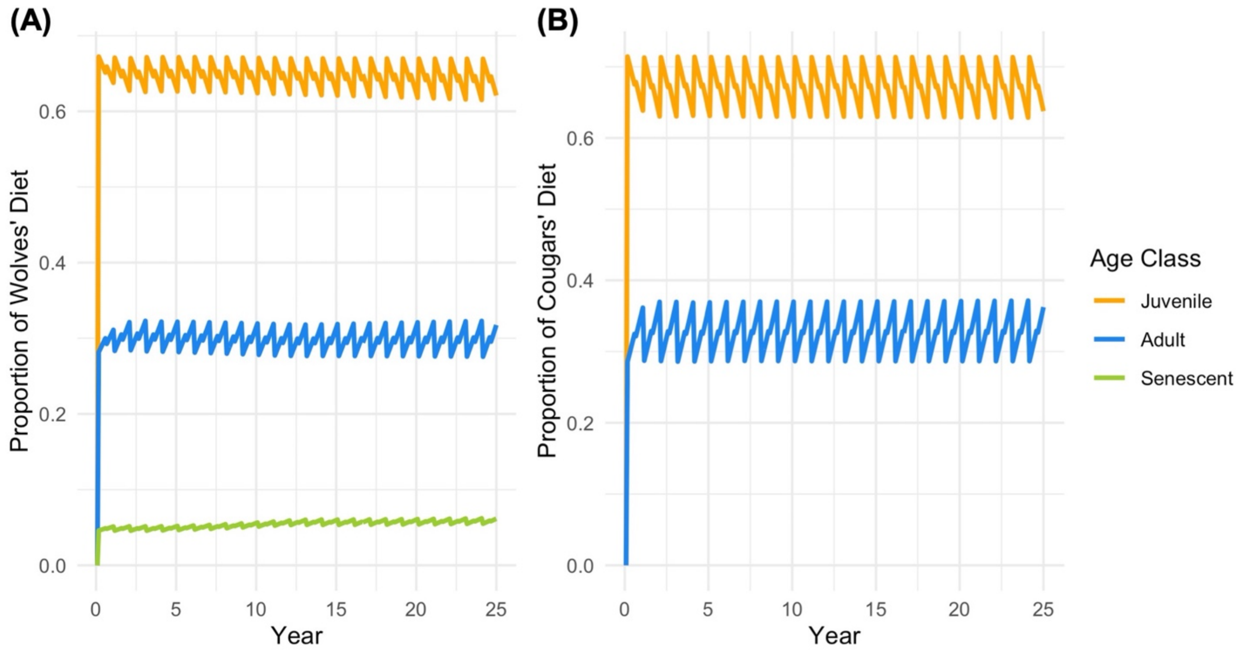

Figure S10. Model output of the proportion of the (A) wolf and (B) cougar population's diet over the 25-year simulation by age class (orange = juvenile, blue = adult, green = senescent). Age classes for cougars are broader (adult = senescent) in order to compare with empirical data.

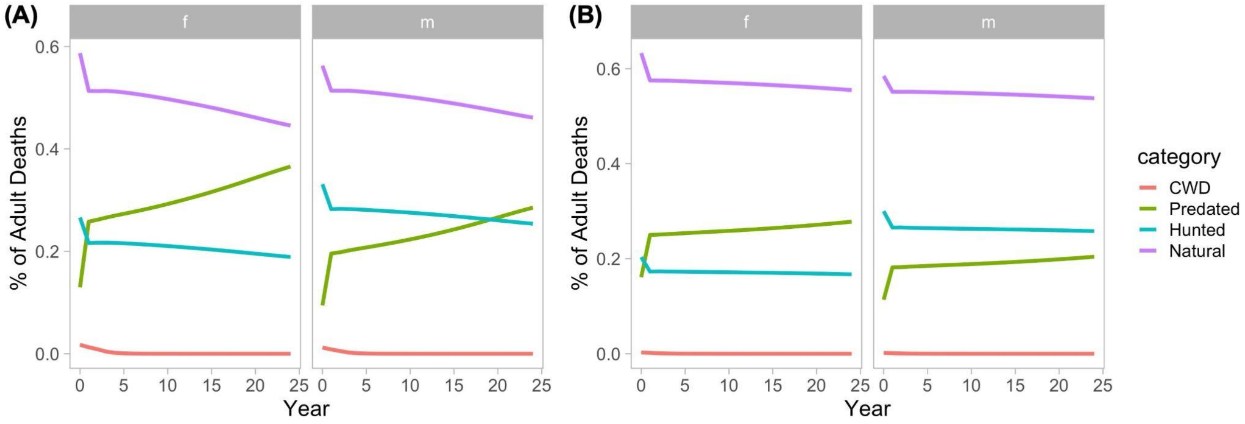

Figure S11. Adult (A) elk and (B) mule deer deaths partitioned by cause: CWD (red), predated (green), hunted (teal), and natural (purple), and sex (left panels f = female, right panels m = male). We note that the proportion of mortality attributed to predation for adult elk and mule deer plausibly fall within approximately 10-40% (Bishop et al. 2005, Bender et al. 2007, Evans et al. 2006, Brodie et al. 2013, Forrester and Wittmer 2013), and "Natural" mortality refers all mortality from sources other than CWD, predation, and human hunting.

REFERENCES

Almberg, E. S., P. C. Cross, C. J. Johnson, D. M. Heisey, and B. J. Richards. (2011). Modeling routes of chronic wasting disease transmission: Environmental prion persistence promotes deer population decline and extinction. *PLoS ONE* 6:e19896.

Barber-Meyer, S. M., L. D. Mech, and P. J. White. (2008). Elk Calf Survival and Mortality Following Wolf Restoration to Yellowstone National Park. *Wildlife Monographs*:1–30.

Bender, L. C., and B. D. Hoenes. (2018). Age-related fecundity of free-ranging mule deer Odocoileus hemionus Cervidae in south-central New Mexico, USA. *Mammalia* 82:124–132.

Bender, L. C., Lomas, L. A., & Browning, J. (2007). Condition, survival, and cause-specific mortality of adult female mule deer in north-central New Mexico. *Journal of Wildlife Management*, *71*(4), 1118–1124. doi:10.2193/2006-226

Bishop, C. J., Unsworth, J. W., & Garton, E. O. (2005). Mule deer survival among adjacent populations in southwest Idaho. *Journal of Wildlife Management*, *69*(1), 311–321.

Bishop, C. J., White, G. C., Freddy, D. J., Watkins, B. E., & Stephenson, T. R. (2009). Effect of Enhanced Nutrition on Mule Deer Population Rate of Change. *Wildlife Monographs*, *172*, 1–28. doi:10.2193/2008-107

Brodie, J., Johnson, H., Mitchell, M., Zager, P., Proffitt, K., Hebblewhite, M., … White, P. J. (2013). Relative influence of human harvest, carnivores, and weather on adult female elk survival across western North America. *Journal of Applied Ecology*, *50*(2), 295–305. doi:10.1111/1365-2664.12044

Davenport, K. A., B. A. Mosher, B. M. Brost, D. M. Henderson, N. D. Denkers, A. v. Nalls, E. McNulty, C. K. Mathiason, and E. A. Hoovera. (2018). Assessment of chronic wasting disease prion shedding in deer saliva with occupancy modeling. *Journal of Clinical Microbiology* 56:1–14.

Eacker, D. R., M. Hebblewhite, K. M. Proffitt, B. S. Jimenez, M. S. Mitchell, and H. S. Robinson. (2016). Annual elk calf survival in a multiple carnivore system. *Journal of Wildlife Management* 80:1345–1359.

Evans, S. B., L. D. Mech, P. J. White, and G. A. Sargeant. 2006. Survival of adult female elk in Yellowstone following wolf restoration. *Journal of Wildlife Management* 70:1372–1378.

Forrester, T. D., and H. U. Wittmer. (2013). A review of the population dynamics of mule deer and black-tailed deer Odocoileus hemionus in North America. *Mammal Review* 43:292–308.

Fox, K. A., J. E. Jewell, E. S. Williams, and M. W. Miller. (2006). Patterns of PrPCWD accumulation during the course of chronic wasting disease infection in orally inoculated mule deer (*Odocoileus hemionus*). *Journal of General Virology* 87:3451–3461.

Hoy, S. R., MacNulty, D. R., Metz, M. C., Smith, D. W., Stahler, D. R., Peterson, R. O., & Vucetich, J. A. (2021). Negative frequency-dependent prey selection by wolves and its implications on predator–prey dynamics. *Animal Behaviour*, *179*, 247-265.

Hoy, S. R., D. R. MacNulty, D. W. Smith, D. R. Stahler, X. Lambin, R. O. Peterson, J. S. Ruprecht, and J. A. Vucetich. (2020). Fluctuations in age structure and their variable influence on population growth. *Functional Ecology* 34:203–216.

Husseman, J. S., D. L. Murray, G. Power, C. Mack, C. R. Wenger, H. Quigley Husseman, J. S. Husseman, and D. L. Murray. (2003). Assessing differential prey selection patterns between two sympatric large carnivores. *OIKOS* 101:591–601.

Johnson, D. H. (1980). The comparison of usage and availability measurements for evaluating resource preference. *Ecology* 61:65–71.

Joly, D. O., M. D. Samuel, J. A. Langenberg, J. A. Blanchong, C. A. Batha, R. E. Rolley, D. P. Keane, and C. A. Ribic. (2006). Spatial epidemiology of chronic wasting disease in Wisconsin white-tailed deer. *Journal of Wildlife Diseases* 42:578–588.

Kunkel, K. E., T. K. Ruth, D. H. Pletscher, and M. G. Doorknocker. (1999). Winter prey selection by wolves and cougars in and near Glacier National Park, Montana. Source: The Journal of Wildlife Management 63:901–910.

Lele, S. R., Merrill, E. H., Keim, J., & Boyce, M. S. (2013). Selection, use, choice and occupancy: Clarifying concepts in resource selection studies. *Journal of Animal Ecology*, 82(6), 1183–1191. doi:10.1111/1365-2656.12141

Lubow, B. C., and B. L. Smith. 2004. Population dynamics of the Jackson Elk Herd. *Journal of Wildlife Management* 68:810–829.

MacNulty, D. R., D. R. Stahler, T. Wyman, J. Ruprecht, L. M. Smith, M. T. Kohl, and D. W. Smith. (2020). Population dynamics of northern Yellowstone elk after wolf reintroduction. Pages 184–199 *in* D. W. Smith, D. R. Stahler, and D. R. MacNulty, editors. Yellowstone Wolves: Science and Discovery in the World's First National Park. University of Chicago Press.

Mech, L. D., and S. Barber-Meyer. (2015). Yellowstone wolf (*Canis lupus*) density predicted by elk (*Cervus elaphus*) biomass. *Canadian Journal of Zoology*:499–502.

Metz, M. C., Smith, D. W., Stahler, D. R., MacNulty, D. R., & Hebblewhite, M. (2020). Wolf predation on elk in a multi-prey environment. In D. W. Smith, D. R. Stahler, & D. R. MacNulty (Eds.), Yellowstone Wolves: Science and Discovery in the World's First National Park (pp. 169–183). University of Chicago Press.

Metz, M. C., Smith, D. W., Vucetich, J. A., Stahler, D. R., & Peterson, R. O. (2012). Seasonal patterns of predation for gray wolves in the multi-prey system of Yellowstone National Park. *Journal of Animal Ecology*. doi:10.1111/j.1365-2656.2011.01945.x

Miller, M. W., & Conner, M. M. (2005). Epidemiology of chronic wasting disease in free-ranging mule deer: Spatial, temporal, and demographic influences on observed prevalence patterns. *Journal of Wildlife Diseases*, 41(2), 275–290. doi:10.7589/0090-3558-41.2.275

Miller, M. W., Runge, J. P., Holland, A. A., & Eckert, M. D. (2020). Hunting pressure modulates prion infection risk in mule deer herds. *Journal of Wildlife Diseases*, 56(4), 781–790. doi:10.7589/jwd-d-20-00054

Miller, M. W., Swanson, H. M., Wolfe, L. L., Quartarone, F. G., Huwer, S. L., Southwick, C. H., & Lukacs, P. M. (2008). Lions and prions and deer demise. *PLoS ONE*, 3(12). doi:10.1371/journal.pone.0004019

Miller, M. W., Williams, E. S., McCarty, C. W., Spraker, T. R., Kreeger, T. J., Larsen, C. T., & Tom Thorne, E. (2000). Epizootiology of chronic wasting disease in free-ranging cervids in Colorado and Wyoming. *Journal of Wildlife Diseases*, 36(4), 676–690. doi:10.7589/0090-3558-36.4.676

Monteith, K. L., Bleich, V. C., Stephenson, T. R., Pierce, B. M., Conner, M. M., Kie, J. G., & Bowyer, R. T. (2014). Life-history characteristics of mule deer: Effects of nutrition in a variable environment. *Wildlife Monographs*, (186), 1–62. doi:10.1002/wmon.1011

Moore, S. J., Vrentas, C. E., Hwang, S., Greenlee, M. H. W., Nicholson, E. M., & Greenlee, J. J. (2018). Pathologic and biochemical characterization of PrP Sc from elk with PRNP polymorphisms at codon 132 after experimental infection with the chronic wasting disease agent. *BMC Veterinary Research*, 14(1), 1–11.

Mule Deer Working Group 2019. (2019). *2019 Range-wide status of black-tailed and mule deer*. Boise, Idaho, USA.

Mysterud, A., & Edmunds, D. R. (2019). A review of chronic wasting disease in North America with implications for Europe. *European Journal of Wildlife Research*, 65(2). doi:10.1007/s10344-019-1260-z

Plummer, I. H., Wright, S. D., Johnson, C. J., Pedersen, J. A., & Samuel, M. D. (2017). Temporal patterns of chronic wasting disease prion excretion in three cervid species. *Journal of General Virology*, 98(7), 1932–1942. doi:10.1099/jgv.0.000845

Potapov, A., Merrill, E., Pybus, M., Coltman, D., & Lewis, M. A. (2013). Chronic wasting disease: Possible transmission mechanisms in deer. *Ecological Modelling*, 250, 244–257. doi:10.1016/j.ecolmodel.2012.11.012

Robinson, S. J., Samuel, M. D., Johnson, C. J., Adams, M., & McKenzie, D. I. (2012). Emerging prion disease drives host selection in a wildlife population. *Ecological Applications*, 22(3), 1050–1059. doi:10.1890/11-0907.1

Ruth, T. K., Buotte, P. C., & Hornocker, M. G. (2019). Yellowstone cougars: ecology before and during wolf restoration. University Press of Colorado.

Samuel, M. D., & Storm, D. J. (2016). Chronic wasting disease in white-tailed deer: Infection, mortality, and implications for heterogeneous transmission. *Ecology*, 97(11), 3195–3205. doi:10.1002/ecy.1538

Stewart, K. M., Bowyer, R. T., Dick, B. L., Johnson, B. K., & Kie, J. G. (2005). Density-dependent effects on physical condition and reproduction in North American elk: An experimental test. *Oecologia*, *143*(1), 85–93. doi:10.1007/s00442-004-1785-y

Storm, D. J., Samuel, M. D., Rolley, R. E., Shelton, P., Keuler, N. S., Richards, B. J., & van Deelen, T. R. (2013). Deer density and disease prevalence influence transmission of chronic wasting disease in white-tailed deer. *Ecosphere*, 4(1), 1–14. doi:10.1890/ES12-00141.1

Tallian, A., Smith, D. W., Stahler, D. R., Metz, M. C., Wallen, R. L., Geremia, C., … MacNulty, D. R. (2017). Predator foraging response to a resurgent dangerous prey. *Functional Ecology*, *31*(7), 1418–1429. doi:10.1111/1365-2435.12866

Tamgüney, G., Miller, M. W., Wolfe, L. L., Sirochman, T. M., Glidden, D. v., Palmer, C., … Prusiner, S. B. (2009). Asymptomatic deer excrete infectious prions in faeces. *Nature*, 461(7263), 529–532. doi:10.1038/nature08289

Varley, N., & Boyce, M. S. (2006). Adaptive management for reintroductions: Updating a wolf recovery model for Yellowstone National Park. *Ecological Modelling*, 193(3–4), 315–339. doi:10.1016/j.ecolmodel.2005.09.001

White, G. C., Garrott, R. A., Bartmann, R. M., Carpenter, L. H., & Alldredge, A. W. (1987). Movements of Female Mule Deer in Northwest Colorado. *Journal of Wildlife Management*, *51*(3), 634–643.

Williams, E. S. (2005). Chronic wasting disease. *Veterinary Pathology*, 42(5), 530–549. doi:10.1354/vp.42-5-530

Williams, Elizabeth S., Miller, M. W., Kreeger, T. J., Kahn, R. H., & Thorne, E. T. (2002). Chronic wasting disease of deer and elk: A review with recommendations for management. *Journal of Wildlife Management*, 66(3), 551. doi:10.2307/3803123

Wilmers, C. C., Metz, M. C., Stahler, D. R., Kohl, M. T., Geremia, C., & Smith, D. W. (2020). How climate impacts the composition of wolf-killed elk in northern Yellowstone National Park. *Journal of Animal Ecology*, 89(6), 1511–1519. doi:10.1111/1365-2656.13200

Wright, G. J., Peterson, R. O., Smith, D. W., & Lemke, T. O. (2006). Selection of Northern Yellowstone elk by gray wolves and hunters. *Journal of Wildlife Management*, *70*(4), 1070–1078. doi:10.2193/0022-541x(2006)70[1070:sonyeb]2.0.co;2
