# Supplementary material for: Examination of the interaction between age‐specific predation and chronic disease in the Greater Yellowstone Ecosystem
Source: J Anim Ecol. 2022 Jan 27;91(7):1373–84. doi: 10.1111/1365-2656.13661 (PMC9912199; doi:10.1111/1365-2656.13661)
Supplement: Supplementary file 2 — Supplementary Material [file JANE-91-1373-s001.pdf]

Supporting Information Appendix 2: Model Dynamics and Sensitivity

Examination of the interaction between age-specific predation and chronic disease in the Greater Yellowstone Ecosystem

Ellen E. Brandell, Paul C. Cross, Douglas W. Smith, Will Rogers, Nathan L. Galloway, Daniel MacNulty, Daniel R. Stahler, John Treanor, Peter J. Hudson

TRANSMISSION MODELS

Our CWD model can be altered so that the 10 CWD disease stages have differential transmission. In the main text, we used a late-stage transmission whereby later CWD stages (8-10) had increased transmission compared to early stages (1-7; Fig. 1A, Fig. S12A). Without strong empirical evidence on the quantity of prions shed throughout an infection, we can formulate other plausible alternatives for how transmission rates may change as CWD progresses. We developed three types of transmission models describing how prion shedding changes as CWD progresses: late stage (Fig. S12A), linear (Fig. S12B), and equal (Fig. S12C).

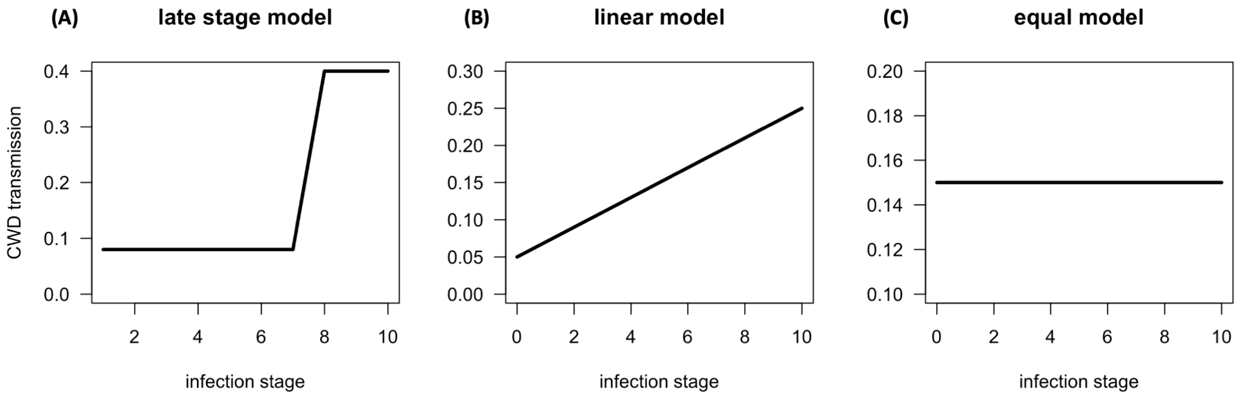

Figure S12. Plot displaying CWD transmission models with different functional forms: (A) late-stage, (B) linear, or (C) equal.

Combinations of predation & transmission models

In the main text, we used the exponential predation model and the late-stage transmission model (Fig. S2A, S12A), but we also explored other combinations (listed in Table S4). To retain similar transmission values across models, for a cougar-deer system, we set β = 0.073 for the null model, β = 0.029, *slope* = 0.01 for the linear, and β = 0.028, M = 7 for the late-stage transmission model. Predation parameters were held constant across all models during comparisons but varied to assess sensitivities (approximately: c = 0.01, r = 0.2, K = 2, P = 10-100 (initial P = 40, N = 8000).

Table S4. Combinations of the predation and transmission models explored in this Supplement. The 'exponential-late' combination was used for simulations in the main text.

Model name	Predation model	Transmission model
null	equal	equal
linear	linear	linear
exponential-linear	exponential	linear
exponential-late	exponential	late-stage

Predators delayed and reduced CWD prevalence and maintained a viable prey population most effectively with the exponential-late model followed by the linear model, although the exponential-linear and linear models behaved very similarly. Transmission rate and predation rate took approximately the same form in the exponential-linear and linear models, which is why resulting prevalence and abundance were nearly equivalent. The exponential-linear and linear models, while effective at removing CWD, tended to result in lower prey abundance than the exponential-late model.

When the kill rate was elevated, the exponential-late and linear models removed CWD the fastest, and the prey population settled to a larger size than the exponential-linear or null models, in that order. Under high selection, exponential prey selection also promoted larger prey populations because of the lower removal rates of uninfected and mildly infected individuals that reproduce (offspring are considered uninfected).

The null model resulted in the highest CWD prevalence, and often, severe population declines from a combination of predation and CWD-induced mortality. Increasing kill rate reduced CWD prevalence under the null model, but also reduced the prey population to the greatest extent compared to the other models. This occurs because predators do not differentiate between uninfected and infected prey, and transmission rates are equivalent across all infection stages – thus CWD is not controlled, and predation and disease are largely additive sources of mortality.

In summary, combinations of linear transmission, late-stage transmission, linear predation, and exponential predation all behaved similarly under moderate predation pressure, but differences were apparent when selection on infecteds was varied. Additionally, different transmission parameters can exacerbate differences among transmission and predation combinations. For instance, as CWD transmission rate increases, the ability of predators to remove CWD under the linear model continued to exceed that of the exponential-linear model because infection and removal were better matched. Given current knowledge about CWD transmission and predation habits, we concluded that exponential predation and late-stage transmission models best represented reality; we recognize, however, that different functional forms can generate different outcomes.

MODEL DYNAMICS & OUTPUT

Here we demonstrate model dynamics, outputs, and diagnostics using an approximate cougar-mule deer system under two scenarios: (1) CWD outbreak occurs under low predation pressure, and (2) CWD outbreak is controlled under high predation pressure (Fig. S13-S15).

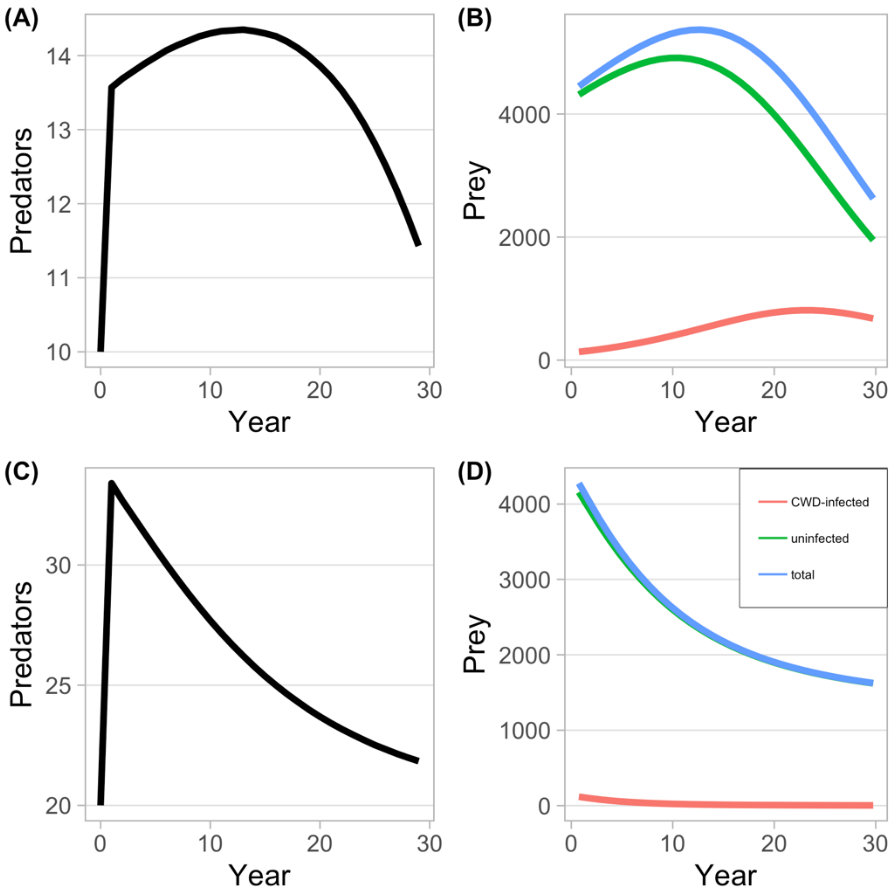

Figure S13. Counts of predators (A, C) and prey (B, D) through time for two types of simulations: (A, B) predators are ineffective at controlling CWD (r = 0.1, K = 0.8), and (C, D) predators successfully control CWD invasion and populations equilibrate (r = 0.3, K = 2); the system represents a cougar-deer system. Prey counts are colored by CWD-infected (red), uninfected (green), and total (blue).

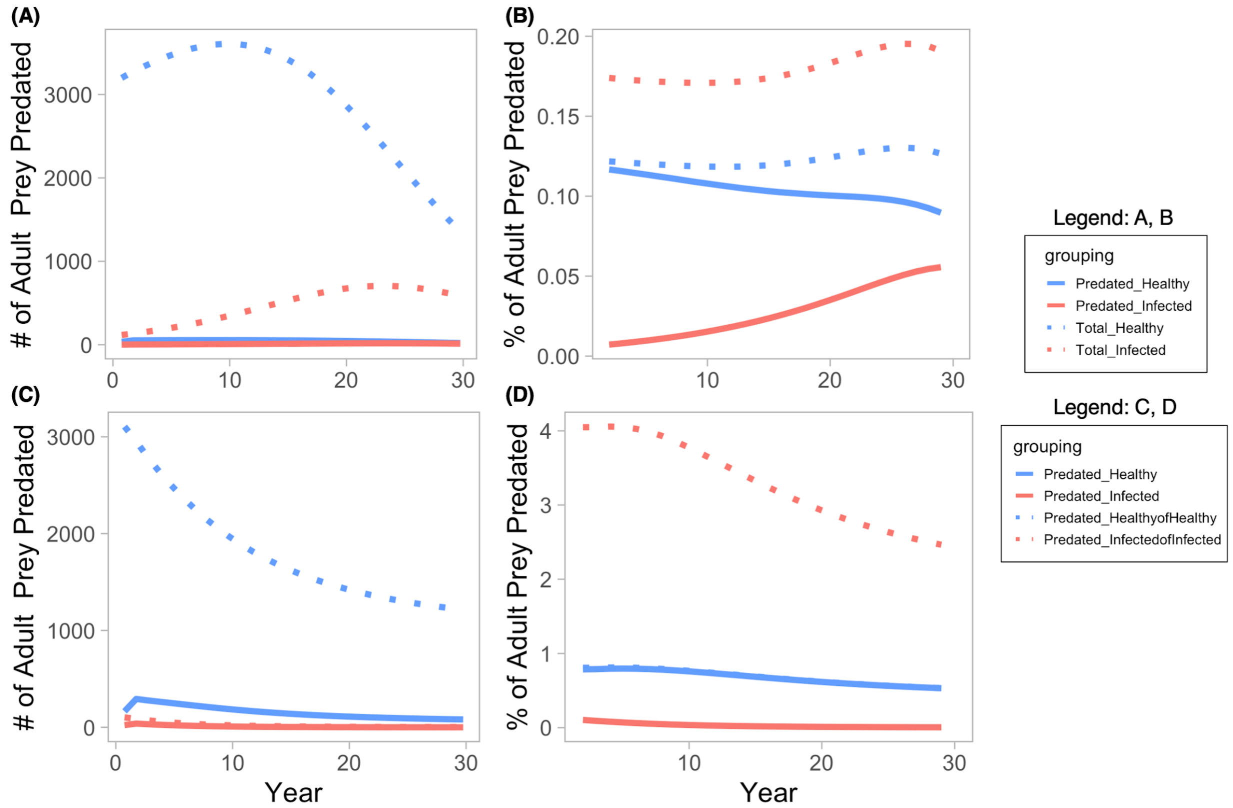

Figure S14. Predation patterns from simulations in which (A, B) predators are ineffective at controlling CWD and (C, D) predators successfully control CWD invasion; the system represents a cougar-deer system from the same simulations as Figure S13. (A, C) The number of adult prey killed by predators, grouped by: healthy killed out of the entire deer population (blue line) and CWD-infected killed out the entire deer population (red line). Dashes are the population size of healthy (blue) and infected (red) deer for comparison. (B, D) The percentage of adult mortalities attributed to predators, grouped by: healthy killed out of the entire deer population (blue line), CWD-infected killed out of the entire deer population (red line), healthy killed out of the healthy deer population (blue dashes), and infected killed out of the infected deer population (red dashes).

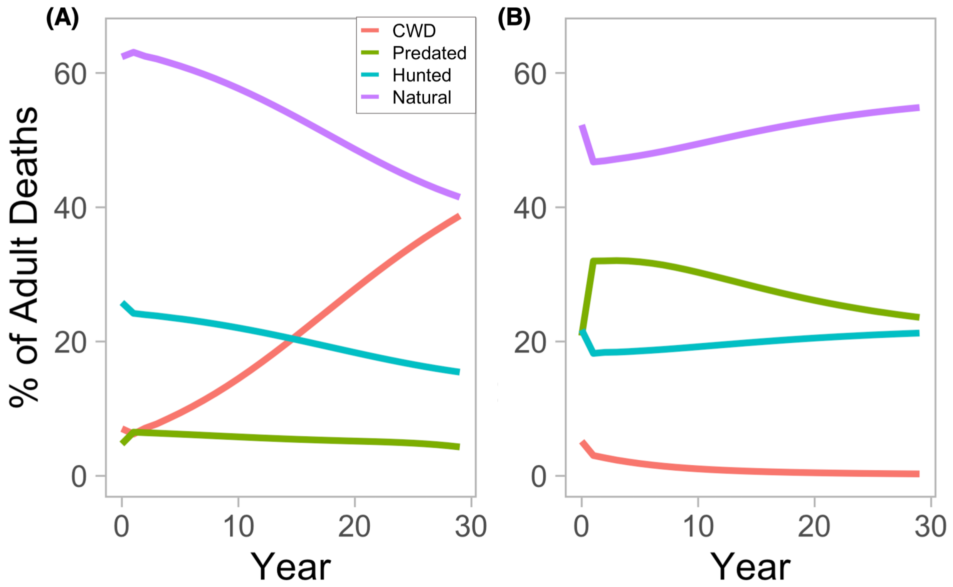

Figure S15. Adult elk deaths partitioned by cause: CWD (red), predated (green), hunted (teal), and natural (purple). Simulations include (A) predators are ineffective at controlling CWD and (B) predators successfully control CWD invasion; the system represents a cougar-deer system from the same simulations as Figure S13.

MODEL SENSITIVITY & PARAMETER INTERACTIONS

To assess model sensitivity, we fixed well-known parameters and varied parameters of interest. We were particularly interested in host-CWD parameters: transmission (β) and rate of CWD progression (ρ), as well as plastic predation habits: selection for infected prey (r), maximum per capita kill rate (K), and baseline selection on adult prey ($b_{i=juvenile,\ j=0}$ versus $b_{i=adult,\ j=0}$).

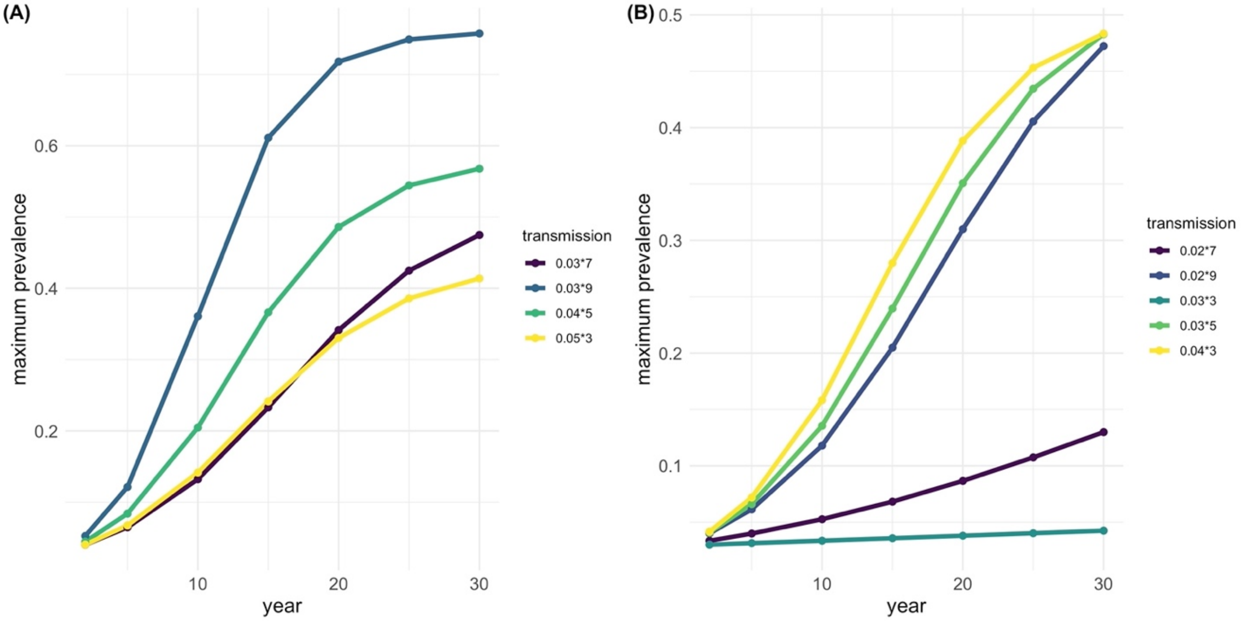

Figure S16. Maximum CWD prevalence reached over a 30-year simulation for different potential combinations of early transmission rates (β) and the multiplier for late-stage transmission (M), for (A) deer and (B) elk. Legends display $\beta*M$ combinations.

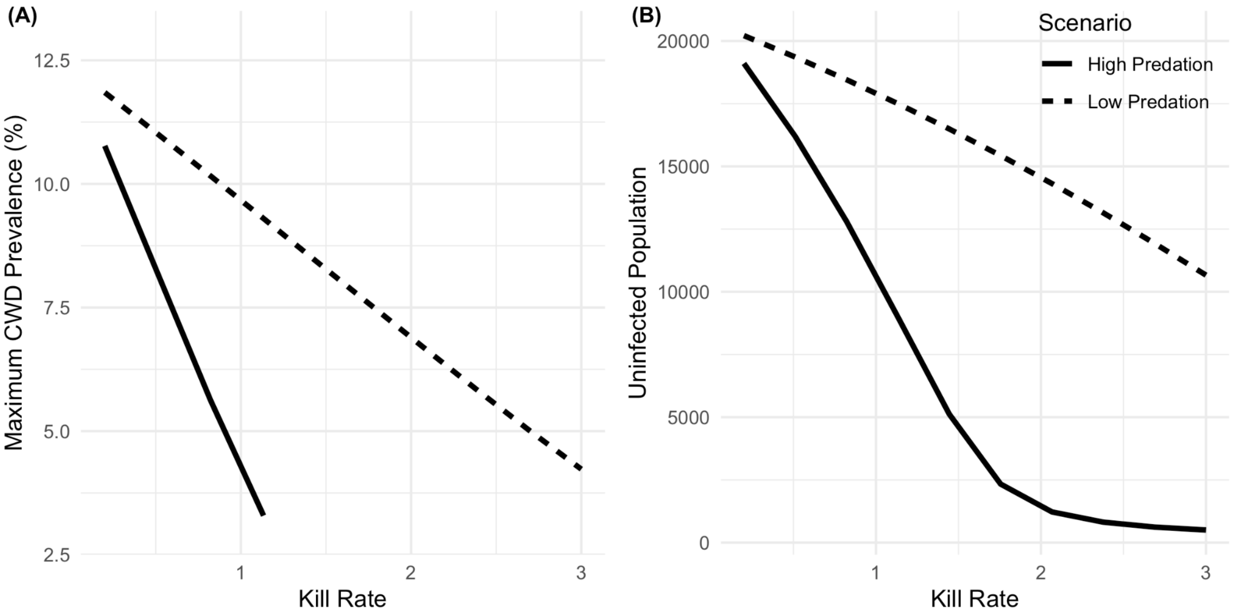

Figure S17. The effect of maximum per capita kill rate *K* on (A) maximum CWD prevalence reached and (B) uninfected host abundance at year 20, under two predation scenarios (r=0.25, P_{wolf} low=20, P_{wolf} high=60).

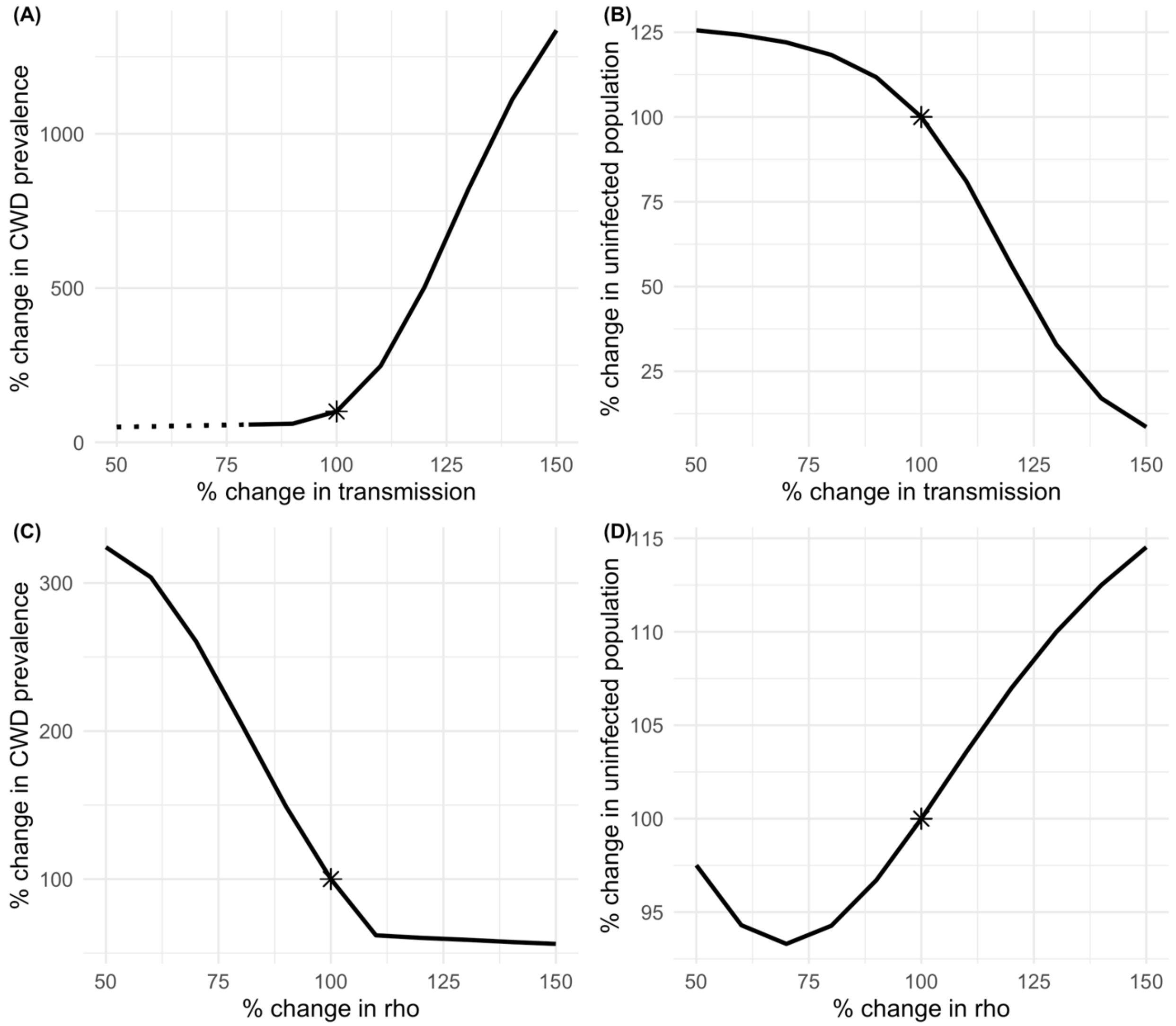

Figure S18. Sensitivity of (A,C) maximum CWD prevalence and (B,D) uninfected elk population size to changes in (A,B) early transmission rate (β 0.013–0.039, M = 5) and (C,D) the rate of CWD progression (ρ = 0.14-0.42, or ~5.8–1.9 years). Asterisks denote values used in simulations as the reference (Table S1); parameter values were incrementally changed (by 10%) from 50% to 150% of the reference value, and changes in maximum CWD prevalence and uninfected elk population size were calculated as percent differences from the results of simulations with the reference parameters. Dashes in (A) denote simulations where maximum CWD prevalence was the initial prevalence (i.e., prevalence declined following introduction). Simulations were run for 20 years with initial prevalence ~2.5%, N = 8000, P_{wolves}=40, r=0.25, K=1.

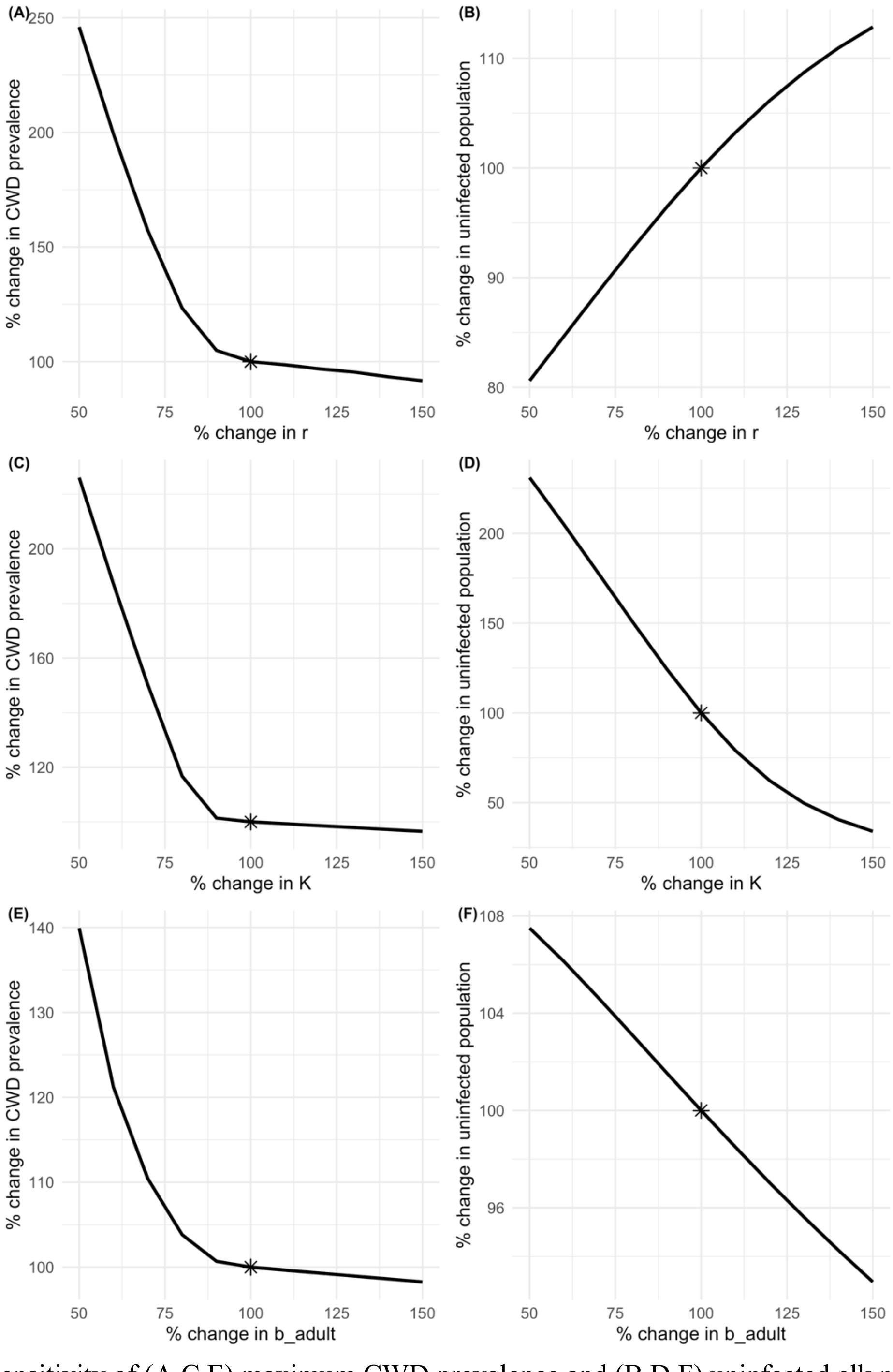

Figure S19. Sensitivity of (A,C,E) maximum CWD prevalence and (B,D,F) uninfected elk population size to changes in (A,B) selection for infected individuals r (selection for stage 10 CWD 3-24x greater than uninfected individuals in the same age class), (C,D) maximum kill rate K (0.75-2.25), and (E,F) selection for adults $b_{i=adult,senescent}$ (ranging from 1/6 to 1/2 that of juvenile selection). Asterisks denote values used in simulations as the reference (Table S1); parameter values were incrementally changed (by 10%) from 50% to 150% of the reference value, and changes in maximum CWD prevalence and uninfected elk population size were calculated as percent differences from the results of simulations with the reference parameters. Simulations were run for 20 years with initial prevalence ~2.5%, N = 8000, P_{wolves}=40, r=0.25, K=1.5.

In terms of host-CWD parameters, maximum CWD prevalence and uninfected host abundance are more sensitive to the rates CWD transmission (Fig. S18A, B) than CWD progression (Fig. S18C, D). Early transmission rates within ~20% of the β reference value (β=0.026) result in similar dynamics such that the epidemic is controlled by year 20, but above ~0.026 CWD prevalence increases dramatically, and the host population steeply declines from higher rates of disease-induced mortality. Maximum CWD prevalence reached in the 20-year simulation is 4.66% for the reference parameter value (Fig. S18A asterisk) and prevalence increases to a maximum of 62.24% when β is increased by 50% (β=0.039) – a 13-fold increase. At this β, the uninfected host population is nearly wiped out (Fig. S19B). When early transmission was below $\beta \approx 0.018$, CWD prevalence declined following introduction and could not be maintained (dashes Fig. S18A).

The effect of changes in CWD progression rate ρ on prevalence is intuitive: as CWD progression slows (i.e., time from infection to death increases, left of 100% reference point), CWD prevalence increases because hosts survive for longer with their infections and are responsible for more transmission events (Fig. S18C). The effects of CWD progression rate on uninfected host population size is interesting and slightly counter-intuitive. At the slowest rate of progression (nearly 6 years median time from infection to death), the host population increases because the effect of CWD approaches the average lifespan of the deer, and most reproduction (i.e., prior to senescence) has already occurred. Then, as CWD progress speeds up (i.e., time from infection to death decreases, right of 100% reference point), hosts die before they are responsible for a significant amount of transmission, and CWD cannot persist at high levels; this allows the host population to escape disease-induced mortality and increase in size. However, in the middle values of ρ, hosts live long enough to transmit the infection to others but also have a few offspring – there likely exists a balance between disease-induced mortality and host birth rate where both the pathogen and the host population are maintained.

Maximum CWD prevalence reached in a 20-year simulation drops drastically as selection for infected individuals relative to uninfected individuals increases (Fig. S19A), kill rate increases (Fig. S19C), and as predators increase their selection for young and senescent adults greater than that of juveniles (Fig. S19E; y-intercepts Fig. 1B; $b_{i=juvenile,\, j=0} = 1$; $b_{i=adult,\, j=0} = b_{i=senescent,\, j=0}$; note this is different from infected adult selection in Fig. 3). However, for all three parameters investigated in Fig. S19, maximum CWD prevalence does not appreciably decline as parameters increase 50% greater than the reference value (asterisks to far right x-axis). CWD prevalence is more sensitive to changes in parameters r and K than b (range of y-axes). These findings suggests that changes in individual predation parameters may not be sufficient to eradicate CWD.

There is a trade-off between reducing CWD prevalence and maintaining a relatively large uninfected host population. As selection for infected individuals relative to uninfected individuals increases (Fig. S19B), kill rate increases (Fig. S19D), and predators increase their selection for young and senescent adults greater than that of juveniles (Fig. S19F), host populations decline. In contrast to changes in CWD prevalence (Fig. S19A, C, E), healthy host abundance continues to decline as parameter values increase, although there might be stabilization at very high kill rates and selection (>150% reference value of K=1.5 and r=0.25). This is also reflected in Fig. S17 and suggests a potential low equilibrium point with low/no CWD present and <50% population abundance compared to the reference.

SENSITIVITY TO FUNCTIONAL AND NUMERICAL RESPONSES

There is uncertainty about the shape of predator numerical and functional responses in terms of the relationship with prey density (e.g., rate of increase, inflection point, asymptote value). This relationship can be defined as: linear (Type I), asymptotic (Type II), or sigmoidal (Type III) (Fig. S20). Typically, the numerical and functional responses are directly linked in a system of equations (e.g., Real 1977, equations 3-6), but here, the functional response occurs on a shorter time scale – monthly – compared to the numerical response which occurs annually. In short, we assume the predator population is stable within a year, but fluctuations in prey density determine the predator population the next year (Eq. 5). Each month (the time step of the model), predators remove prey based on their selection (Eq. 2) and the abundance of each age and infection class *ij* (Eq. 3), as well as the predator's maximum per capita kill rate (Eq. 4). Therefore, numerical and functional responses still exert a strong influence on predator-prey dynamics and abundances, even as separate equations in our model framework.

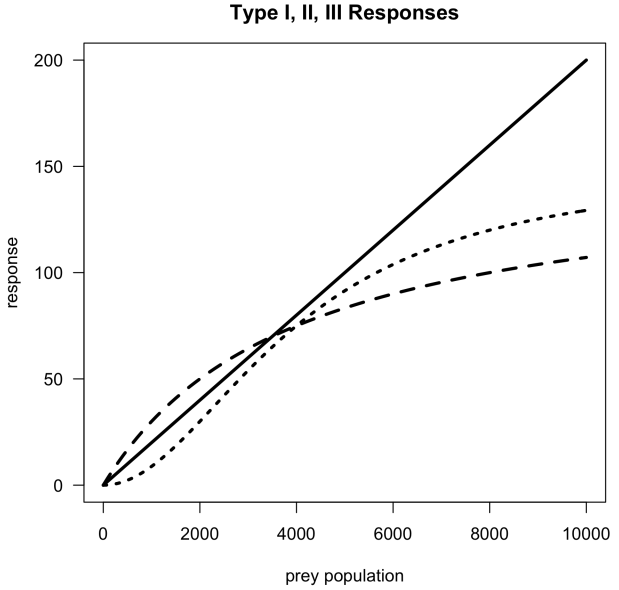

Figure S20. The potential relationships between prey density and response, where 'response' can be predator kill rate (functional response) or predator population growth rate (numerical response). Type I = solid, Type II = dashes, and Type III = dots.

Generally, Type II and III are considered the most probable shapes for a predator's functional response because predators are limited by the time it takes to hunt, subdue, and consume their prey, thus Type I is unlikely. Empirically, Type II and Type III are challenging to distinguish because they require adequate sample sizes at low prey densities, and confident estimates of kill rates and prey abundance. Type III is assumed to occur when predators can switch their selection to a different prey species when their preferred prey is scarce, and at low densities, prey may be better at occupying refugia from predators. Type II is considered destabilizing as predators can drive their prey to extinction whereas Type III is stabilizing.

Both Type II and Type III functional responses have statistical support in the Yellowstone wolf-elk system (Becker et al. 2008, Metz et al. 2020). Additionally, functional responses may differ by season (Metz et al. 2020). If CWD were present in the Yellowstone system, it is plausible to assume that predators would

switch from killing their typically preferred prey species, to other infected, vulnerable cervids. Based on this information, we cannot select a functional response with complete certainty at present, but Type II or III are both likely thus we feel confident in the realism of our simulations.

Wolf numerical response has not been studied with the rigor of the functional response. Again, Type II and Type III are the most probable in the Greater Yellowstone Ecosystem because wolf populations are intrinsically regulated via territoriality and aggression (Cubaynes et al. 2014, Cassidy et al. 2015, Smith et al. 2015). Additionally, the y-intercept of the numerical response is above zero in populations with immigration or when there is more than one prey species available. In addition to reproduction, the numerical response has been used to describe an increase or decrease in predator density in general, such as predator aggregation during the prey birth pulse. Both Type II and Type III numerical responses are supported in the Yellowstone wolf-elk system (Metz et al. 2020). Without elk, Metz et al. (2020) estimated that 30 wolves would live in northern Yellowstone (using a Type III curve) - we typically set our minimum wolf population size to 20-30 in our simulations.

We conducted a sensitivity analysis whereby we assessed wolf and elk population trajectories and size over a 20-year simulation as functional and numerical responses are altered (Type II, Type III; Table S5), as well as the corresponding inflection points (ϕ, δ). Predation parameters were held constant across all models during comparisons but varied to assess sensitivities. Predation and transmission models were exponential and late-stage, respectively, corresponding to the main text.

Table S5. Combinations of functional and numerical responses explored in this Supplement. The FR3_NR2 was used for simulations in the main text.

**Model name**	**Functional response**	**Numerical Response**
FR2_NR2	Type II	Type II
FR2_NR3	Type II	Type III
FR3_NR2	Type III	Type II
FR3_NR3	Type III	Type III

Predator-prey relationships

Models with a Type III numerical response resulted in more turbulent wolf population trajectories, often overshooting their abundance in year 20 to a greater extent than using a Type II numerical response. With a Type III functional response, the elk population decreased at an increasing rate then stabilized (i.e., backwards S-shaped); with a Type II functional response, the elk population trajectory was highly variable depending on parameters selected and form of the numerical response. Thus a Type III functional response was more stabilizing on elk populations than Type II.

When kill rate was moderate to high, the FR3_NR3 model often resulted in the lowest CWD prevalence, and the quickest reduction in CWD prevalence (Fig. S21). However, if predators were not selective enough, the FR3_NR3 model did not remove all CWD and prevalence began to increase again after year ~10-15. This is a result of a reduction in predator abundance and predation rate at lower elk abundance due to the sigmoidal curves.

Under most plausible scenarios, all models produced qualitatively similar outcomes, except FR2_NR2, which was least effective at controlling CWD. For example, combinations of functional and numerical responses were generally consistent about whether CWD was eradicated or not by year 20. If CWD was eradicated, the time to eradication tended to be consistent across all models at 15-20 years. Type II and III responses are notably different when prey abundance is low, however, this mainly occurs during a CWD outbreak when CWD-induced mortality surpasses predator-induced mortality, in which case the population is very unlikely to recover regardless.

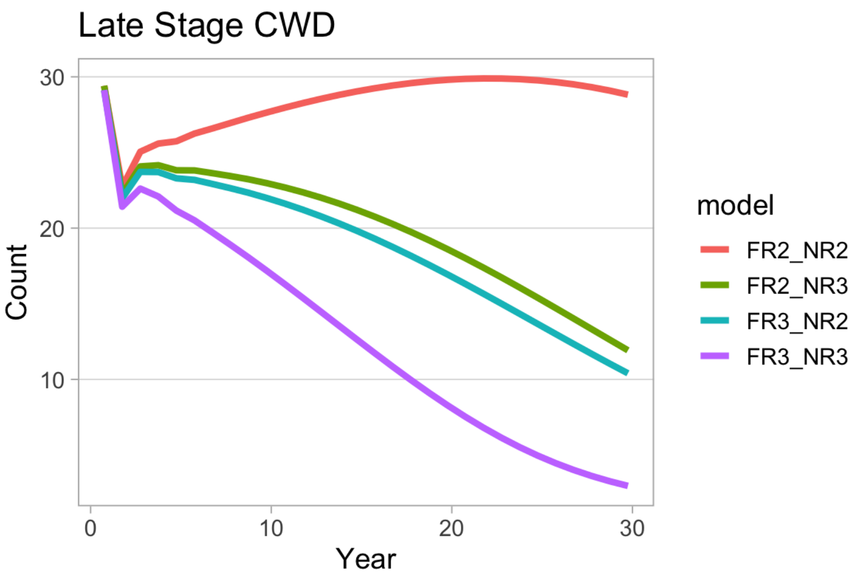

Figure S21. Count of late-stage CWD infections (*j*=8-10) through time given different models of functional and numerical responses (colors). Simulations represent a hypothetical wolf-elk system.

To summarize, the CWD prevalence, prey abundance, and predator abundance trajectories differed within a simulation depending on the form of the functional and numerical responses, however, the long-term results (15+ years) were very similar. As expected, FR2_NR2 and FR3_NR3 were the most dissimilar to each other such that FR3_NR3 was more likely to result in CWD elimination and lower prey abundance. Additionally, the form of the functional response had a larger influence on CWD prevalence, prey abundance, and predator abundance trajectories than the numerical response. We used the FR3_NR2 model in the main text, which was less extreme and tended to result in relatively stable predator and prey populations.

Inflection points

Here we individually varied the inflection points for the functional (ϕ) and numerical (δ) responses (from 500-8,000) and examined the effect on CWD prevalence, prey abundance, and predator abundance trajectories (Fig. S22), using a wolf-elk system. We used the FR3_NR2 model, late-stage transmission, and exponential predation, as in the main text.

As the numerical response inflection point increased from 500-8,000, ~stable wolf abundance nearly doubled (i.e., population growth slowed; Fig. S22A). Uninfected elk abundance did not drastically change within a decade, and population growth was stunted at high values of the numerical response inflection point because wolf populations were able to grow over a larger range of prey population sizes (Fig. S22B).

Under different numerical responses, CWD prevalence increased or decreased steadily within approximately ±3% of initial prevalence (Fig. S22C).

Wolf populations were less variable to changes in functional response than numerical response, by definition. Still, as functional response inflection value increased, the predator abundance trajectory changed from negative to positive, and growth slowed (Fig. S22D). CWD prevalence was very sensitive to functional responses, ranging from CWD removal to over 8% by year 20 as the inflection value increased (Fig. S22F). We suspect these patterns occur because, with larger inflection points, predation rates are lower for a greater range of prey population sizes, thus CWD transmission rate surpasses predation rate and the infection spreads. Uninfected host abundance was also variable, and at the upper range was suppressed by the growing CWD outbreak (Fig. S22E).

To summarize, CWD prevalence and host abundance were more sensitive to changes in functional response inflection points (Fig. S22D-F) than numerical response (Fig. S22A-C). Exact inflection points are very challenging to estimate using field data, but fortunately, results were very similar over the plausible range for Yellowstone data (inflection ~2000-3500).

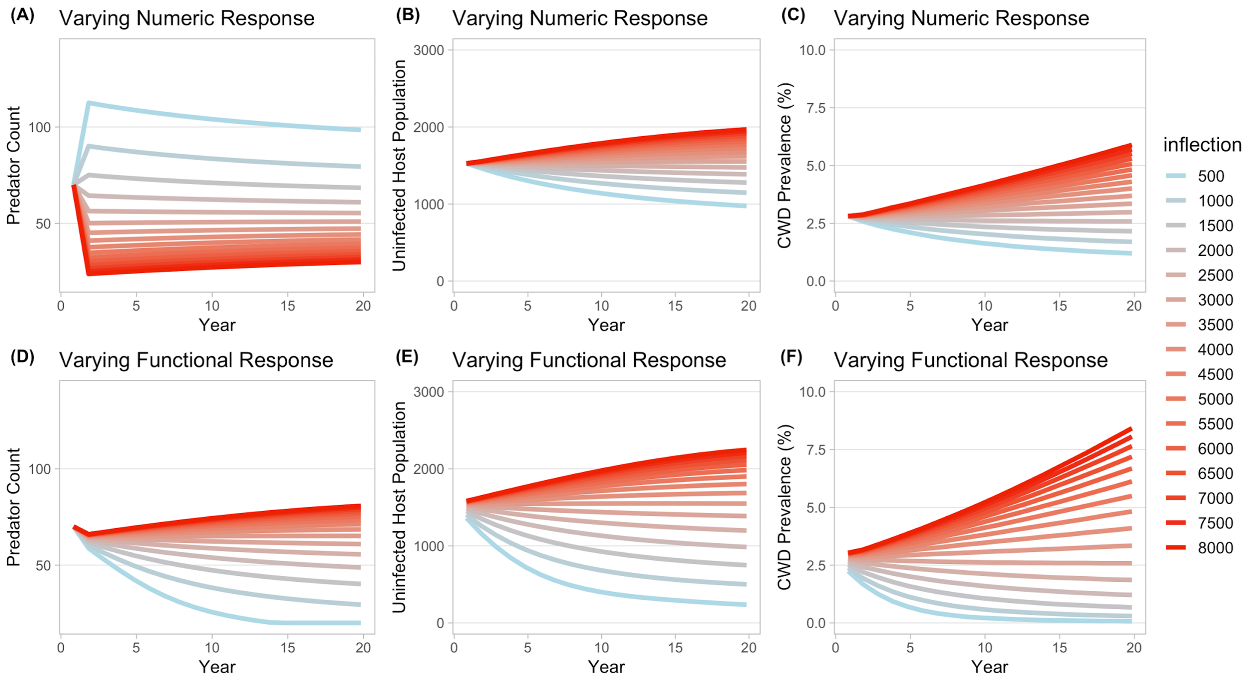

Figure S22. Sensitivity of (A,D) predator abundance, (B,E) uninfected host abundance, and (C,F) CWD prevalence (% infected) to changes in the inflection point value for (A-C) numerical and (D-F) functional response. Simulations represent a hypothetical wolf-elk system ($r = 0.3$, $K = 0.5$, $P = 20$-150, initial $P = 70$, $N = 1500$), and inflection point values ranged from 500 (light blue) to 8000 (red).

REFERENCES

Becker, M. S., R. A. Garrott, P. J. White, R. Jaffe, J. J. Borkowski, C. N. Gower, and E. J. Bergman. (2008). Wolf kill rates: predictably variable? Pages 339–369 *in* R. Garrott, P. J. White, and F. Watson, editors. The Ecology of Large Mammals in Central Yellowstone. Elsevier Science & Technology.

Cassidy, K. A., D. R. MacNulty, D. R. Stahler, D. W. Smith, and L. D. Mech. 2015. Group composition effects on aggressive interpack interactions of gray wolves in Yellowstone National Park. *Behavioral Ecology* 26:1352–1360.

Cubaynes, S., D. R. Macnulty, D. R. Stahler, K. A. Quimby, D. W. Smith, and T. Coulson. (2014). Density-dependent intraspecific aggression regulates survival in northern Yellowstone wolves (*Canis lupus*). *Journal of Animal Ecology* 83:1344–1356.

Metz, M. C., Smith, D. W., Stahler, D. R., MacNulty, D. R., & Hebblewhite, M. (2020). Wolf predation on elk in a multi-prey environment. In D. W. Smith, D. R. Stahler, & D. R. MacNulty (Eds.), Yellowstone Wolves: Science and Discovery in the World's First National Park (pp. 169–183). University of Chicago Press.

Metz, M. C., Smith, D. W., Vucetich, J. A., Stahler, D. R., & Peterson, R. O. (2012). Seasonal patterns of predation for gray wolves in the multi-prey system of Yellowstone National Park. *Journal of Animal Ecology*. doi:10.1111/j.1365-2656.2011.01945.x

Real, L. A. (1977). The Kinetics of Functional Response. *The American Naturalist*, 111(978), 289–300. Retrieved from https://www.jstor.org/stable/2460064

Smith, D. W., Metz, M. C., Cassidy, K. A., Stahler, E. E., McIntyre, R. T., Almberg, E. S., & Stahler, D. R. (2015). Infanticide in wolves: seasonality of mortalities and attacks at dens support evolution of territoriality. *Journal of Mammalogy*, 96(6), 1174–1183. doi:10.1093/jmammal/gyv125
