# Supplementary material for: Examination of the interaction between age‐specific predation and chronic disease in the Greater Yellowstone Ecosystem
Source: J Anim Ecol. 2022 Jan 27;91(7):1373–84. doi: 10.1111/1365-2656.13661 (PMC9912199; doi:10.1111/1365-2656.13661)
Supplement: Supplementary file 3 — Supplementary Material [file JANE-91-1373-s003.pdf]

Supporting Information Appendix 3: Management Implications

Examination of the interaction between age-specific predation and chronic disease in the Greater Yellowstone Ecosystem

Ellen E. Brandell, Paul C. Cross, Douglas W. Smith, Will Rogers, Nathan L. Galloway, Daniel MacNulty, Daniel R. Stahler, John Treanor, Peter J. Hudson

MANAGEMENT IMPLICATIONS

Some of our assumptions and predation parameters could be validated in the field as CWD invades the Yellowstone ecosystem. Kill rate and composition, prey abundance and demographics, predator abundance, and baseline selection are readily estimable, while other parameters are more challenging, such as selection for infected prey. Understanding predation habits with respect to infection requires estimates of predation rate and CWD prevalence in the prey population (preferably by age and sex class), as well as infection status of individuals predated (i.e., level of severity of infection or time since infection). The empirical effects of predators on CWD invasion and spread will not be evident for years to decades given that CWD progresses slowly, and it has only recently spread into areas inhabited by wolves and grizzly bears. In addition, current assays are limited in scope and availability, often require antemortem tissues, and it is logistically challenging to obtain the large-scale sampling (and resampling) of hosts required for disease monitoring.

To this end, we examined the time it takes for a symptomatic population to accumulate (j = 8-10) either in terms of percent of the population or counts. This may be useful to managers that conduct observational studies to note a certain percentage of symptomatic individuals when monitoring herds, and managers or tourists may detect a symptomatic host quickly after CWD introduction in the Greater Yellowstone Ecosystem where visibility and tourism are high.

As the predator population increases, the accumulation of severely infected hosts (i.e., symptomatic, j = 8-10) is delayed (Fig. S23). Without predators, 3% of deer and elk populations are symptomatic about ten years following CWD introduction at ~2% prevalence. As the number of predators increases, the accumulation of late-stage infections is delayed significantly – for instance, increasing the cougar population from zero to 10 delayed the accumulation of late-stage deer (3%) by a decade. Simply detecting a symptomatic host could occur more quickly after CWD introduction (~5 years, Fig. S23 purple lines). The symptomatic population of deer accumulated more quickly than elk but was also more sensitive to the effects of predation (Fig. S23A vs S23B slope of lines and predator counts).

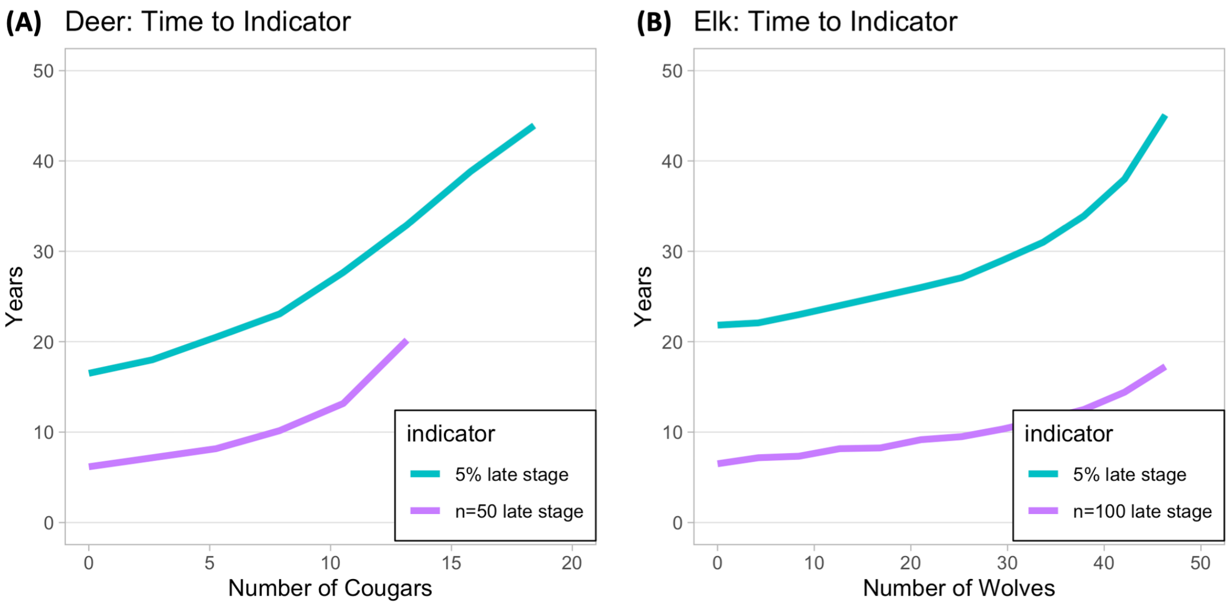

Figure S23. The time it takes (years) for adult late-stage hosts (A: mule deer, B: elk) to build up in the population based on predator abundance (A: cougars, B: wolves, r = 0.2, K_{cougar} = 2, K_{wolf} = 0.7). The accumulation of late-stage hosts is measured as a percentage (5% of total population) or count (deer=50, elk=100). Initial prey abundance corresponded with observations in the northern Yellowstone area: approximately 3000 deer and 7000 elk.

Based on Figures S1 and S23, CWD detection via observations may not be extremely useful in the short-term. One promising alternative is the use of quantitative assays for prions in cervid feces (John et al. 2013, Cheng et al. 2016, Henderson et al. 2017) – prey feces could be collected from predator-killed carcasses and paired with data on fecal prion shedding rates (Tamgüney et al. 2009), to provide information about prey selection by infection severity. Quantitative assays could also be used on pooled fecal samples to detect background CWD prevalence (Ahmed et al. 2020, Michael-Kordatou et al. 2020). Finally, as CWD prions may stay viable through the canid digestive tract for a few days following consumption (Nichols et al. 2015), wolf scats could be collected, tested for prions, and kill rates with respect to CWD status could be used to examine selective predation. Together, these methods could improve our knowledge of CWD presence, infection severity, and prey selection.

An easier monitoring option for Yellowstone National Park could be examining changes in kill composition. Predators might increase their selection for adult prey that are highly vulnerable, and because kill rates and compositions are monitored year-round for both cougars and wolves in Yellowstone National Park, managers might be able to detect a shift in predation rates by age class. Ideally, managers would know the infection status of predator-killed carcasses, but this is extremely challenging or impossible in many field settings, as wolves, cougars, and scavengers remove nearly all biomass from carcasses. It is likely more feasible to increase monitoring of the prey population by deploying GPS collars with mortality sensors and determining cause of death; as follows, differential survival rates stratified by prey age, sex, and infection status can be analyzed to understand predator-prey-pathogen dynamics.

ADDING COUGARS TO THE WOLF-ELK-CWD MODEL

To explore the effects of multiple predators on a prey species, we added cougars into the wolf-elk model. We added cougars to the wolf-elk model, instead of wolves to the cougar-deer model, because elk comprise ~50% of cougar diet in Yellowstone (Yellowstone Cougar Project) whereas deer comprise <10% of wolf diet in Yellowstone (Metz et al. 2012). Cougar predation was implemented in the model framework as a removal of a plausible constant annual percentage of each infection status (3% uninfected, 8% infected). We emphasize that this section is meant to be an exploration of the effects of multiple predators without changing other sources of mortality; in reality, we might expect managers to reduce harvest rates (especially on adult females) when host populations decline.

With moderate wolf pressure (K = 0.5-1.5, r = 0.15-0.35, initial P_{wolf} = 20-50), the addition of cougars significantly reduced CWD outbreak size (40-70%). In fact, through time, CWD prevalence continued to decline in the wolf + cougar model but could increase in the wolf-only model (Fig. S24). However, adding cougars also caused the uninfected elk population to notably decline, demonstrating potential trade-offs of a multi-predator system.

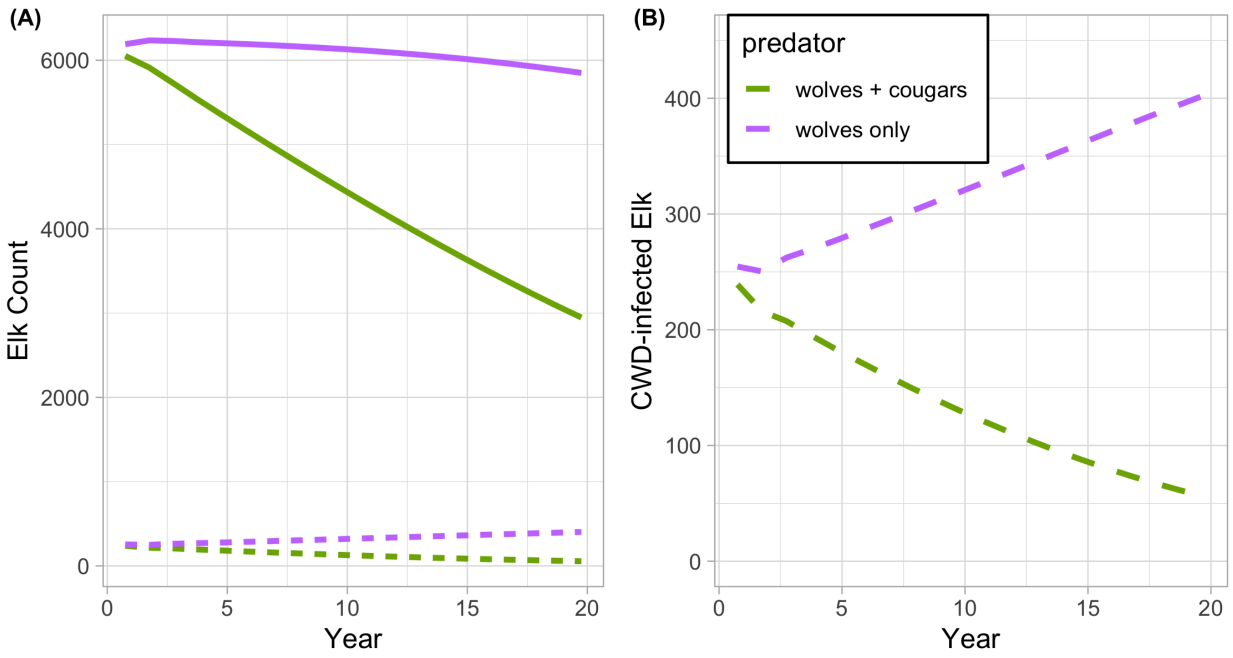

Figure S24. Plots showing elk count with wolf predators only (purple) and wolves and cougars (green), showing uninfected elk (solid lines) and infected elk (dashes). Plot (B) is a zoom-in of the infected elk counts shown in (A).

REFERENCES

Ahmed, W., N. Angel, J. Edson, K. Bibby, A. Bivins, J. W. O'Brien, P. M. Choi, M. Kitajima, S. L. Simpson, J. Li, B. Tscharke, R. Verhagen, W. J. M. Smith, J. Zaugg, L. Dierens, P. Hugenholtz, K. v. Thomas, and J. F. Mueller. (2020). First confirmed detection of SARS-CoV-2 in untreated wastewater in Australia: A proof of concept for the wastewater surveillance of COVID-19 in the community. *Science of the Total Environment* 728:138764.

Cheng, Y. C., S. Hannaoui, T. R. John, S. Dudas, S. Czub, and S. Gilch. (2016). Early and non-invasive detection of chronic wasting disease prions in elk feces by real-time quaking induced conversion. *PLoS ONE* 11:1–18.

Henderson, D. M., Tennant, J. M., Haley, N. J., Denkers, N. D., Mathiason, C. K., & Hoover, E. A. (2017). Detection of chronic wasting disease prion seeding activity in deer and elk feces by real-time quaking-induced conversion. *Journal of General Virology*, *98*(7), 1953–1962. doi:10.1099/jgv.0.000844

John, T. R., H. M. Schätzl, and S. Gilch. (2013). Early detection of chronic wasting disease prions in urine of pre-symptomatic deer by real-time quaking-induced conversion assay. *Prion* 7:253–258.

Metz, M. C., Smith, D. W., Vucetich, J. A., Stahler, D. R., & Peterson, R. O. (2012). Seasonal patterns of predation for gray wolves in the multi-prey system of Yellowstone National Park. *Journal of Animal Ecology*. doi:10.1111/j.1365-2656.2011.01945.x

Michael-Kordatou, I., Karaolia, P., & Fatta-Kassinos, D. (2020). Sewage analysis as a tool for the COVID-19 pandemic response and management: the urgent need for optimised protocols for SARS-CoV-2 detection and quantification. *Journal of Environmental Chemical Engineering*, 8(5), 104306. doi:10.1016/j.jece.2020.104306

Nichols, T. A., Fischer, J. W., Spraker, T. R., Kong, Q., & VerCauteren, K. C. (2015). CWD prions remain infectious after passage through the digestive system of coyotes (*Canis latrans*). *Prion*, *9*(5), 367–375. doi:10.1080/19336896.2015.1086061

Tamgüney, G., Miller, M. W., Wolfe, L. L., Sirochman, T. M., Glidden, D. v., Palmer, C., … Prusiner, S. B. (2009). Asymptomatic deer excrete infectious prions in faeces. *Nature*, 461(7263), 529–532. doi:10.1038/nature08289
